# Non-essential genes form the hubs of genome scale protein function and environmental gene expression networks in *Salmonella enterica* serovar Typhimurium

**DOI:** 10.1186/1471-2180-13-294

**Published:** 2013-12-17

**Authors:** Jesper T Rosenkrantz, Henk Aarts, Tjakko Abee, Matthew D Rolfe, Gitte M Knudsen, Maj-Britt Nielsen, Line E Thomsen, Marcel H Zwietering, John E Olsen, Carmen Pin

**Affiliations:** 1 Department of Veterinary Disease Biology, University of Copenhagen, Stigbøjlen 4, 1870 Frederiksberg, C, Denmark; 2 Centre for Infectious disease control, National Institute for Public Health, PO box 1, 3720 BA Bilthoven, The Netherlands; 3 Wageningen University and Research Centre, Laboratory of Food Microbiology, P.O. Box 17, 6700 AA Wageningen, Netherlands; 4 Institute of Food Research, Norwich Research Park, Norwich NR4 7UA, UK; 5 National Food Institute, Danish Technical University, Soelvtofts Plads, 2800 Kgs. Lyngby, Denmark; 6 Present address: DANSTEM Laboratory, University of Copenhagen, Blegdamsvej 3B, 2200 Copenhagen, N, Denmark

## Abstract

**Background:**

*Salmonella* Typhimurium is an important pathogen of human and animals. It shows a broad growth range and survives in harsh conditions. The aim of this study was to analyze transcriptional responses to a number of growth and stress conditions as well as the relationship of metabolic pathways and/or cell functions at the genome-scale-level by network analysis, and further to explore whether highly connected genes (hubs) in these networks were essential for growth, stress adaptation and virulence.

**Results:**

*De novo* generated as well as published transcriptional data for 425 selected genes under a number of growth and stress conditions were used to construct a bipartite network connecting culture conditions and significantly regulated genes (transcriptional network). Also, a genome scale network was constructed for strain LT2. The latter connected genes with metabolic pathways and cellular functions. Both networks were shown to belong to the family of scale-free networks characterized by the presence of highly connected nodes or hubs which are genes whose transcription is regulated when responding to many of the assayed culture conditions or genes encoding products involved in a high number of metabolic pathways and cell functions.

The five genes with most connections in the transcriptional network (*wraB, ygaU, uspA, cbpA and osmC*) and in the genome scale network (*ychN, siiF (STM4262), yajD, ybeB and dcoC*) were selected for mutations, however mutagenesis of *ygaU* and *ybeB* proved unsuccessful. No difference between mutants and the wild type strain was observed during growth at unfavorable temperatures, pH values, NaCl concentrations and in the presence of H_2_O_2._ Eight mutants were evaluated for virulence in C57/BL6 mice and none differed from the wild type strain. Notably, however, deviations of phenotypes with respect to the wild type were observed when combinations of these genes were deleted.

**Conclusion:**

Network analysis revealed the presence of hubs in both transcriptional and functional networks of *S.* Typhimurium. Hubs theoretically confer higher resistance to random mutation but a greater susceptibility to directed attacks, however, we found that genes that formed hubs were dispensable for growth, stress adaptation and virulence, suggesting that evolution favors non-essential genes as main connectors in cellular networks.

## Background

*Salmonella enterica* serovar Typhimurium (*S.* Typhimurium) is an important intestinal pathogen of man and animals [1]. It normally invades the host in the intestine leading to a self-limiting gastro-enteritis [2], but it may also cause a systemic disease in which it resides inside professional phagocytic cells [3]. In mice it causes a Typhoid-like disease, and in this model the contribution of many genes to disease is well-characterized [4].

Studies in the closely related bacterium *Escherichia coli* have demonstrated that the transcriptional response to one stress-stimulus overlaps with the response to other stimuli in a highly adaptive manner [5,6], creating an element of cross resistance towards different stress conditions. While the transcriptional responses of *S.* Typhimurium during growth and in response to different environmental stress conditions have also been detailed [7-10], a systematic analysis of how the *S.* Typhimurium responses interact with each other has not been performed.

Network analysis is a powerful tool to analyze interactions between different matrixes [11]. Networks representing widely different things such as social relations [12], molecular biochemical regulation [13,14] and transcriptional responses in bacteria [15] have all been shown to belong to the family of scale-free networks, which are characterized by the presence of hubs, i.e. highly connected nodes [16]. Preferential attachment is a mechanism that explains the scale-free topology, i.e. new nodes link preferentially with the more connected nodes or hubs [16]. Hubs confer an exceptional robustness to networks towards random node failures; however, directed attacks towards hubs theoretically cause a major network disruption [16].

In transcriptional network analysis of bacterial responses to different growth conditions and different functionalities, such hubs would represent genes that are significantly regulated in response to many different conditions or which are involved in many different pathways and cell functions. From an evolutionary point of view it would be risky, if genes that form these connections were indispensable for cell functions, since mutation in one of these genes would then have consequences for the ability of the bacterium to adapt to many different conditions. In the current study we performed network analysis of transcriptional responses of *S.* Typhimurium to a number of growth and stress conditions and of the global functionality of products encoded in the genome. We then analyzed the topology and the functionality of the most connected genes detected in these two networks and demonstrated that highly connected genes indeed were dispensable for growth, stress adaptation and virulence. Hence it appeared that cellular networks of *S.* Typhimurium were not susceptible to attacks directed towards single hubs.

## Results

Transcriptional response to different environmental stresses share many genes, and genes that are up-regulated at one environmental stress condition are not likely to be down-regulated as response to another condition.

We constructed a microarray consisting of 425 carefully selected stress and virulence genes and used this to assess the transcriptional response of *S.* Typhimurium to heat, osmotic, oxidative and acid stress under anoxic and oxic conditions and to non-stressed anoxic conditions. Therefore, our study was not a genome scale transcriptional response analysis but it was focused on the regulation of the 425 genes most relevant for stress response and virulence. The selection of the 425 genes was based in numerous previous experimental evidences compiled in the GenBank database and they represent the most suitable subset of genes to visualize gene expression under stress conditions; however, it is possible that some information could be missing regarding other genes not included in the array design. A total of 283 genes were differentially expressed in response to these environments (Additional file 1: Table S1). Table 1 shows the number of genes up- and down-regulated under each environmental condition and the number of common genes whose regulation was affected in more than one assayed culture condition.

**Table 1:** Number of genes up or down-regulated, detected with the stress and virulence thematic array, under different experimental conditions

		**Heat**		**H**_**2**_**O**_**2**_		**Acid**		**NaCl**	**No stress**	^**a**^**Induced only in this condition**
		**No O**_**2**_	**O**_**2**_	**No O**_**2**_	**O**_**2**_	**No O**_**2**_	**O**_**2**_	**No O**_**2**_	**O**_**2**_	
Heat	No O_2_	+72 & -76*	+51 & -48	+42 & -39	+32 & -36	+44 & -29	+30 & -39	+16 & -17	+23 & -31	4: *sptP, iacP, mgtA, ssaR*
	O_2_		+109 & -88	+58 & -50	+50 & -48	+53 & -40	+49 & -52	+19 & -21	+33 & -37	
H_2_O_2_	No O_2_			+112 & -76	+55 & -46	+59 & -42	+44 & -47	+19 & -23	+20 & -33	10: *fur, folE, sdiA, yicC, cheM, polA, sitA, entD, dsrA, fadA*
	O_2_				+76 & -76	+47 & -42	+47 & -55	+19 & -15	+20 & -39	
Acid	No O_2_					+99 & -71	+59 & -52	+19 & -20	+28 & -27	5: *pmrA (basR), fkpA, pmrF, yhjC, cadB*
	O_2_						+76 & -96	+18 & -21	+19 & -42	
NaCl	No O_2_							+28 & -29	+5 & -14	6: *proX, dps, hilC, ybiI, yciF, yehY*
No stress	No O_2_								+62 & -79	8: *prgI, prgK, hycB, hypE, nfnB, rfaB, rt, prgJ*

*The diagonal of the matrix shows the total number of genes up (+) and down (−) regulated in each condition (underlined). Values in other positions show the number of common genes up (+) and down (−) regulated in both conditions.

^a^Genes induced exclusively under one condition (not affected or repressed under the other conditions).

**Figure 1:**
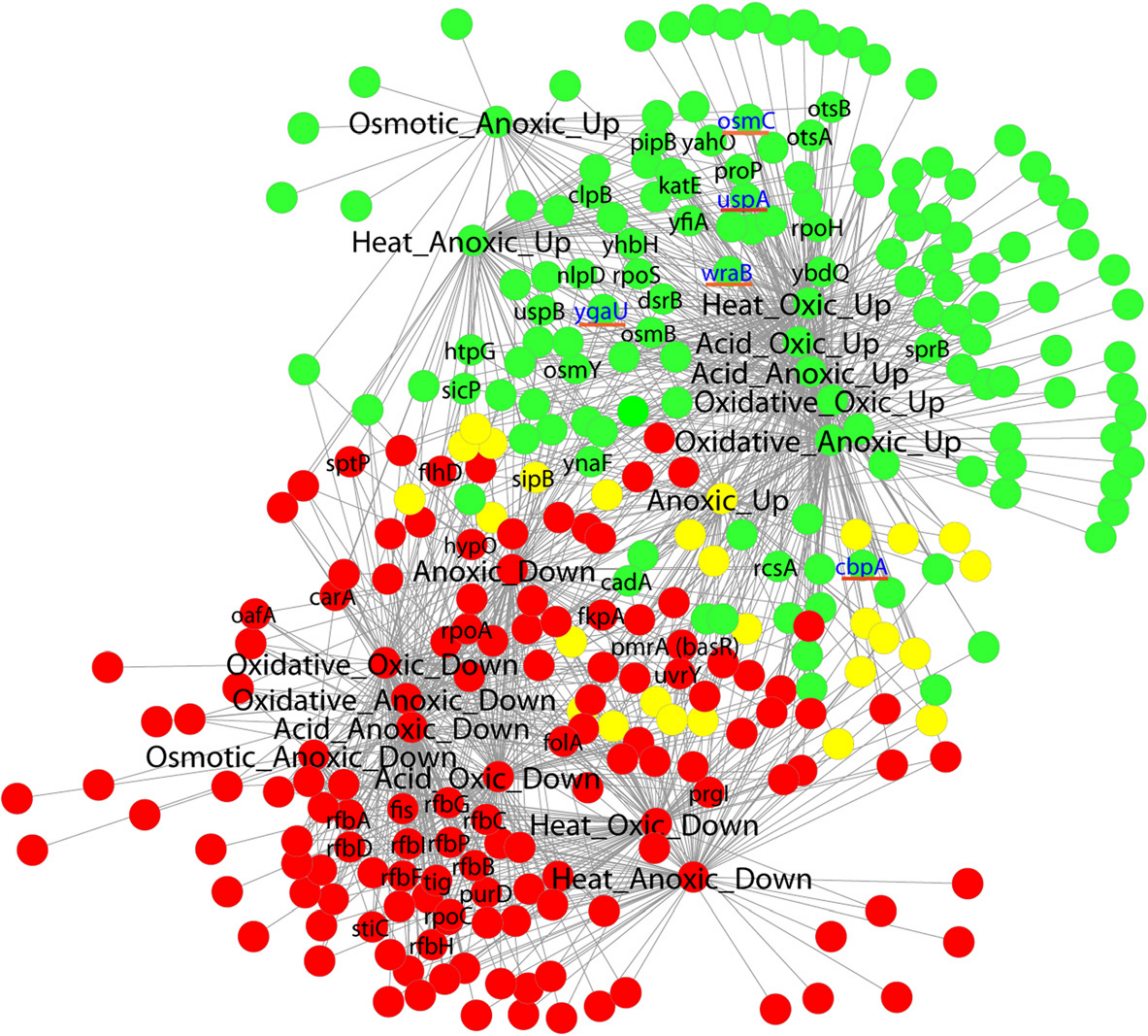
**Network 1 represents those genes included in the stress and virulence thematic microarray that were up(down)-regulated in response to several environmental stresses and anoxic condition.** The bi-partite network connects genes with environmental conditions and regulation pattern. Node colors represent the modules, i.e. highly connected groups of nodes, detected in this network. Gene names added only for highly connected nodes, i.e. hubs with between 4 and 8 links as described in Table S2. The 5 selected hubs to carry out mutations are in blue font and underlined in red.

To analyze the interactions in the transcriptional responses of *S.* Typhimurium, a bipartite network, named Network 1, was constructed by connecting genes with environmental conditions according to expression pattern, i.e. up- or down-regulated (Figure 1). The modularity of this network was analyzed to find patterns of association among environmental stresses. Modularity analysis investigates the existence of communities of highly interconnected nodes in the network that are not connected with other communities. The network modular structure is quantified by the modularity value, Q, which can vary between 0 if no modules are detected and 1, when modularity is at maximum. In practice it has been found that a value above 0.3 is a good indicator of significant community structure in a network [11]. The Q-value for Network 1 was 0.35 and the number of modules detected was 3 (Figure 1). One of the large modules grouped 146 genes that were up-regulated (Figure 1) under the assayed stresses, while the other large module contained 138 genes which were down-regulated. The third module was smaller and included 29 genes with variable expression. This indicates that those genes up- or down-regulated under one environmental stress are not likely to be down- or up-regulated as response to a different environmental stress.

**Figure 2:**
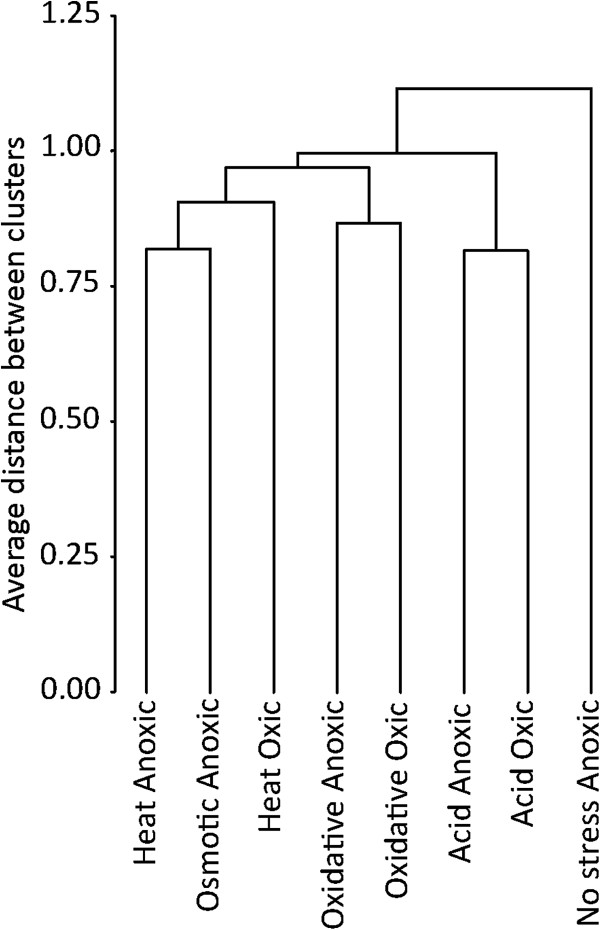
Results of clustering the environmental stresses and anoxic condition according to the associated transcriptional profile observed on the stress and virulence thematic microarray.

As the modular structure indicated, there was a common transcriptional response to several stresses in many genes and no remarkable differences were noticed between stress responses under oxic and anoxic conditions in this respect. Thirty-nine genes were detected as induced under one environmental condition and not induced or repressed under the other conditions (Table 1). All the other detected genes were affected under more than one condition (Table 1). Cluster analysis of the environmental conditions according to their transcriptional profiles revealed that the distance between profiles observed under oxic and anoxic conditions for each stress was sometimes as large as the distance between profiles observed under different stresses (Figure 2). The greatest distance was observed between the transcriptional profile under non-stressed conditions and the profiles observed in stressed cultures. The response to anoxic conditions observed in stressed cultures was different from that observed in non-stressed situations. None of the 10 genes induced only under anoxic conditions in a non- stressed situation was up-regulated in the stressed cultures. Therefore, the stress transcriptional response of many genes was common for different stresses. We targeted to explore those genes that were affected by a large number of stresses and culture conditions.

Network analysis reveals the presence of hubs or highly frequent differentially transcribed genes responding to environmental stresses, growth stages and immobilization

To extend the information contained in Network 1, we constructed Network 2 by adding to Network 1 the transcription patterns associated with the growth stage and immobilization condition as can be found in the original publications [7-9]. In this way, we studied whether the transcription of the 425 genes contained in the microarray used above was affected by the growth stage and immobilization condition.

**Figure 3:**
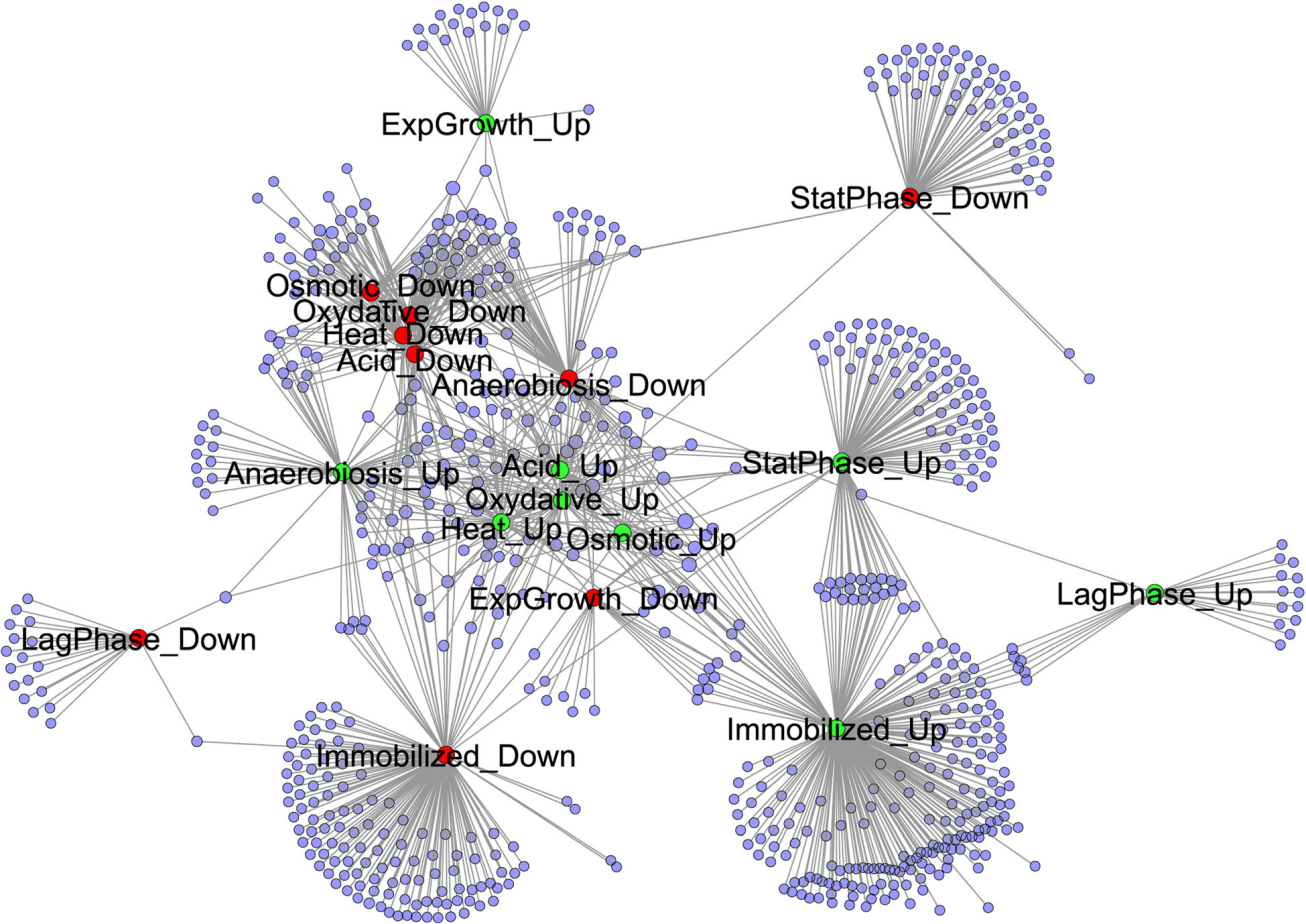
Network 2 is an extension of Network 1 that represents genes up(down)-regulated at various growth stages and immobilization condition together with those responding to several environmental stresses and anoxic condition included in Network 1.

Network 2 (Figure 3) connected genes with environmental stresses, growth stages and immobilization condition according to expression pattern. The layout of the network informed on common up- or down-regulated genes among the tested conditions. The lag period had the most distinctive transcriptional profile with few genes affected under other conditions. However, a small number of genes induced during lag phase were also induced in immobilized cells. The majority of genes down-regulated during lag and in stationary phase were not affected under any other situation. A large number of up-regulated genes in immobilized cultures were also induced in stationary phase. The transcription of several genes in response to environmental stresses was inversely related with their expression during exponential growth. Figure 3 shows that the node representing genes induced during exponential growth was connected with few genes repressed under stressing environments while the node for genes repressed in exponential growth was linked with genes up-regulated in response to stress conditions.

**Figure 4:**
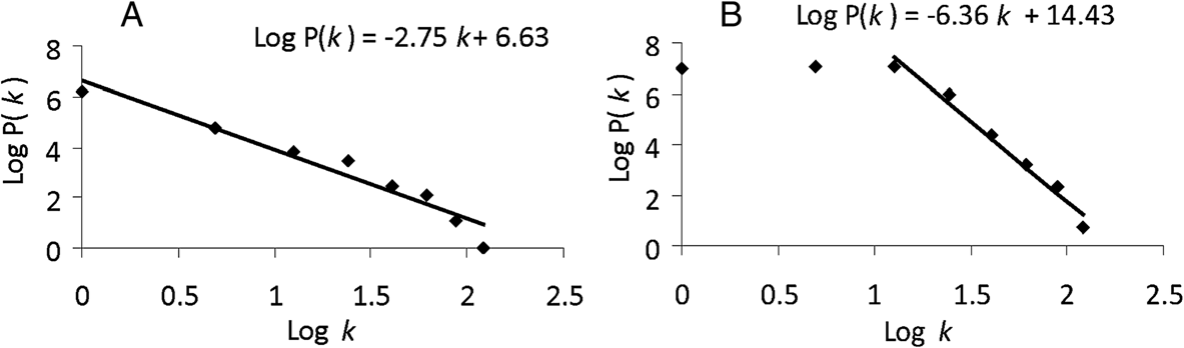
**Nodes degree distribution -P(*k*) represents the probability that the number of links per node is equal to *k*- of the genes connected to environmental stresses, growth stage or immobilization condition in the environmental Network 2 (A) and of the genes connected to metabolic pathways and cellular roles in the *S*. Typhimurium genome scale Network 3 (B).** Distributions followed the power law indicating the existence of highly connected genes or hubs in both networks.

**Figure 5:**
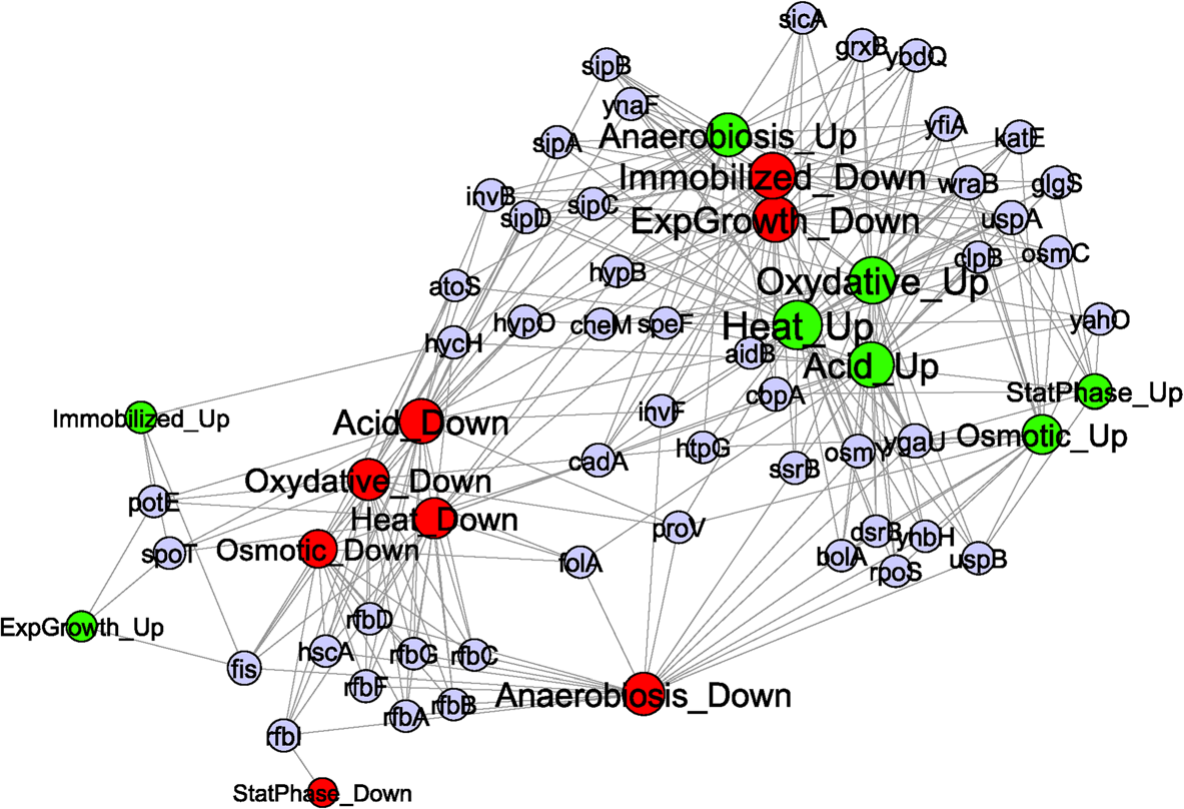
Network 2.1, which is a sub-network from Network 2 including only genes differentially transcribed in the majority of environmental conditions (hubs).

The genes degree (*k*) distribution of the transcriptional response networks decayed as a power law, P(*k*) ~ *k*^–2.7^(Figure 4A), i.e. the network belonged to the family of scale-free networks characterized by the presence of few highly connected genes or hubs corresponding to the genes that were differentially transcribed in many conditions. A list of 54 genes forming hubs in Network 2 is included in supplementary material (Additional file 2: Table S2). Figure 5 shows a sub-network extracted from Network 2 (termed Network 2.1), containing exclusively the 54 genes that formed hubs together with the conditions at which they were differentially transcribed. The transcription of none of these hubs was regulated during the lag phase.

Analysis of the genome scale network for S. Typhimurium shows a scale free topology with hubs formed by genes involved in many metabolic pathways and cellular functions.

To explore the presence of hubs in the genome of *Salmonella*, we looked for genes involved in a large number of cellular functions and metabolic pathways in a genome scale bi-partite network (termed Network 3) constructed for the genome and plasmids of *S.* Typhimurium as previously described [10]. The network was bipartite and thus edges connected two sets of nodes – genes with metabolic pathways and cellular functions. Information was collected from public available resources and databases specified in the Methods section. The total number of nodes in the genome scale network was 5153 of which 4717 were genome and plasmid genes, while the remaining nodes were metabolic pathways and cellular functions. The distribution of the nodes degree (or number of edges belonging to the same node) was estimated independently for genes, metabolic pathways and cellular functions and followed the power law in every case (data not shown). The gene degree distribution was estimated using connections between genes and main functional roles and metabolic pathways only in order to avoid redundancies due to sub-classifications. The tail of the genes degree distribution (*k*) decayed as a power law P(*k*) ~ *k*^-6.4^ indicating the existence of highly connected nodes (Figure 4B). A list of 114 highly connected genes as well as their connections with metabolic pathways and functional roles is included in supplementary material (Additional file 3: Table S3).

### Effect of single deletion of genes forming hubs on the growth and response to environmental stresses of S. Typhimurium

The top five genes in terms of connections to other nodes of the network in Network 2 and Network 4 were selected (Table 2). Single mutants were constructed for eight of these genes in *S.* Typhimurium strain 4/74 (*wraB, uspA, cbpA and osmC* from Network 2 and *ychN, siiF (STM4262), yajD, and dcoC* from Network 4), while mutagenesis of the gene *ygaU* proved unsuccessful in several attempts and mutants of *ybeB* were unstable.

**Table 2:** The highest ranked environmental and functional hubs

**Gene**	**Protein blast**	**Number conditions or functional categories**
**Environmental hubs**		
*ygaU*	LysM domain/BON superfamily protein	8
*osmC*	Putative envelope protein	7
*uspA*	Universal stress protein A	7
*wraB*	NAD(P)H:quinone oxidoreductase, type IV	7
*cbpA*	Curved DNA-binding protein	6
**Functional hubs**		
*ychN*	Putative sulphur reduction protein	8
*siiF*(STM4262)	Putative ABC-type bacteriocin/lantibiotic exporter	8
*yajD*	Hypothetical protein (possible endonuclease superfamily)	7
*ybeB*	Hypothetical protein (possible involved in biosynthesis of extracellular polysaccharides)	7
*dcoC*	Oxaloacetate decarboxylase subunit gamma	7

**Figure 6:**
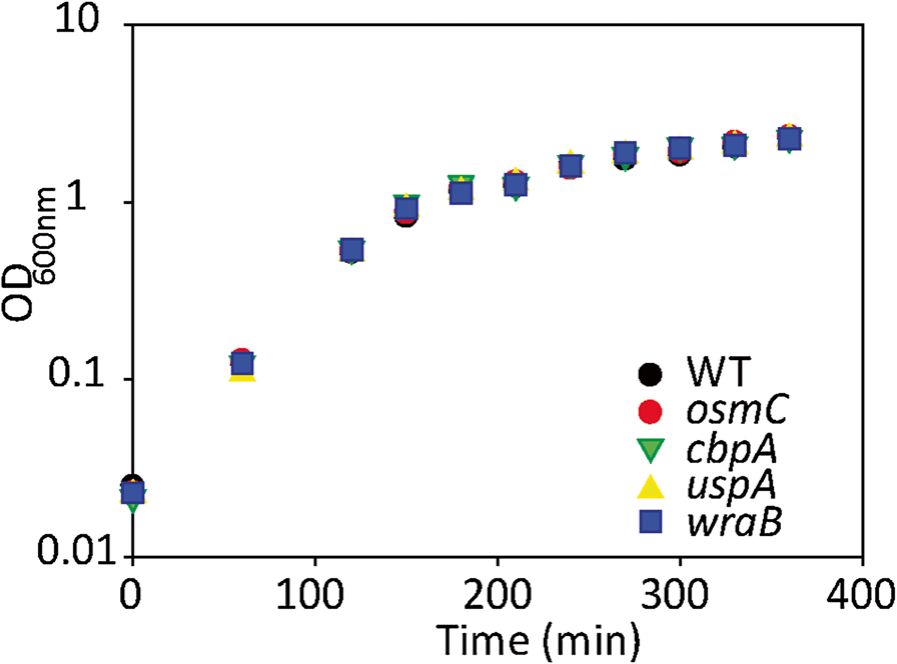
**Growth of wild type and selected mutant strains of *S. *** **Typhimurium deficient in genes identified as environmental hubs in LB at 37°C.**

A summary of growth and stress response phenotypes of these mutants is given in Table 3. All tested mutants grew equally well as the wild type strain in LB broth at 37°C, as illustrated for 4 selected mutants in Figure 6. Mutants were then subjected to a number of growth and stress conditions. As observed for growth at 37°C, mutants did not grow differently from the wild type at 15°C and 44°C, and their growth response to various concentrations of NaCl and different pH values did not differ from that of the wild type strain (Table 3). Furthermore, the analysis of resistance towards H_2_O_2_ did not reveal any difference between wild type and single mutant strains (Table 3).

**Table 3:** **Comparison of the growth/survival response to various environmental conditions of *S. *** **Typhimurium ST4/74 with the response of single and double mutants**

**Strains**	**Description (deletions)**	**Source**	**Temp: 15, 37, 44°C**^**a**^	**NaCl: 2, 4%**	**pH: 5, 9, 10, 11**	**H**_**2**_**O**_**2**_**: 15 mM**
ST4/74	Wild type	Wray [62]				
JTR.446	*osmC*	This study	-	-	-	-
JTR.452	*yajD*	This study	-	-	-	-
JTR.454	*dcoC*	This study	-	-	-	-
JTR.462	wraB*	This study	-	-	-	-
JTR.463	uspA*	This study	-	-	-	-
JTR.464	cbpA*	This study	-	-	-	-
JTR.465	ychN*	This study	-	-	-	-
JTR.466	*siiF*(STM4262)*	This study	-	-	-	-
JTR.472	*uspA/ychN*	This study	-	-	-	-
JTR.473	*uspA/osmC*	This study	-	-	-	-
JTR.474	*uspA/cbpA*	This study	-	-	-	-
JTR.475	*uspA/wraB*	This study	-	-	-	-
JTR.476	*uspA/dcoC*	This study	-	-	-	-
JTR.477	*uspA/yajD*	This study	-	-	-	-
JTR.478	*uspA/siiF*(STM4262)	This study	-	-	-	+
JTR.479	*wraB/yajD*	This study	-	-	-	-
JTR.481	*wraB/ychN*	This study	-	-	-	+
JTR.482	*wraB/osmC*	This study	-	-	-	+
JTR.483	*wraB/dcoC*	This study	-	-	-	+
JTR.484	*wraB/ siiF*(STM4262)	This study	-	-	-	-
JTR.485	*wraB/cbpA*	This study	-	-	-	+
JTR.486	*ychN/cbpA*	This study	-	-	-	-
JTR.487	*ychN/yajD*	This study	-	-	-	+
JTR.489	*ychN/siiF*(STM4262)	This study	-	-	-	-
JTR.490	*ychN/dcoC*	This study	-	-	-	-
JTR.496	*cbpA/yajD*	This study	-	-	-	+
JTR.498	*cbpA/osmC*	This study	-	-	-	+
JTR.499	*cbpA/dcoC*	This study	-	-	-	-
JTR.501	*siiF*(STM4262)*/osmC*	This study	-	-	-	-
JTR.502	*siiF*(STM4262)*/yajD*	This study	-	-	-	-
JTR.503	*siiF*(STM4262)*/cbpA*	This study	-	-	-	-

^a^: List of conditions at which differences were detected. Minus sign denotes no difference between mutant and wild type strain whereas plus sign denotes that the ability to grow or to survive was significantly decreased in mutants.

*Strains used for construction of double mutants.

### Effect of single deletion of genes forming network hubs on the virulence of S. Typhimurium

Virulence characteristics of seven of the eight genes were available from literature and were not repeated in the present investigation. According to literature, strains deficient in *ygaU, uspA, cbpA, ychN, siiF (STM4262)* and *dcoC* were not significantly different from the virulence of the wild type strain [4,17]. The single deletions of *wraB* or *osmC* were even reported to increase the virulence of the mutated strains [4]. Thus, none of these seven genes have been reported to be essential for virulence. Challenge assays in mice were conducted with the *yajD* mutant*.* The deletion of *yajD* proved not to have a significant influence on the outcome of the infection (Table 4).

**Table 4:** Virulence of selected mutant strains

**Strains**	**Description**	^**1**^**CI ± SD**
JTR.452	*yajD*	1.2 ± 0.3
JTR.481	*wraB & ychN*	1.9 ± 0.7*
JTR.482	*wraB & osmC*	0.7 ± 0.2*
JTR.490	*ychN & dcoC*	1.4 ± 0.9
JTR.498	*cbpA & osmC*	1.4 ± 0.3
JTR.499	*cbpA & dcoC*	0.4 ± 0.1*

^1^The competitive index (CI) and its standard deviation (SD) were calculated by dividing the proportion of mutant colonies in the spleen by the proportion of mutant colonies in the inoculum used to infect the mice, as previously reported [75].

*The CI is significantly different from 1, by one-sample t-test, indicating a significant change in the ability of the mutant strain to reside or propagate in mice with respect to the wild type.

### Effect of double mutation of genes forming hubs on growth, stress adaptation and virulence of S. Typhimurium

*S.* Typhimurium shows a high degree of redundancy in metabolic reactions [18], and based on this we decided to test for interactions between gene-products of genes that formed hubs. Twenty-three different double deletion mutants were constructed (Table 3). No difference between wild type and mutated strains was observed during growth at the different temperatures, pH and NaCl concentrations, while the resistance towards H_2_O_2_ was affected for eight of the double knockout mutants (Table 3). This decreased resistance was more often observed when the mutated genes were environmental hubs. From the eight affected double mutants, four of them included the *wraB* environmental hub and three of them were deficient in *cbpA*, which is also an environmental hub. Two of the double mutants deficient in *osmC* (environmental hub)*, ychN* (functional hub) and *yajD* (functional hub) also exhibit a decreased resistance towards H_2_O_2_. (Table 3). Five double mutants were also assessed for virulence. The competition indexes (CI) of these strains are listed in Table 4. The ability of the mutants to propagate in mice was enhanced in one case and reduced in two: The *wraB/ychN* double mutant strain had a significantly increased CI of 1.9, while the values of the CI for the *wraB/osmC* and the *cbpA/dcoC* double mutants were significantly reduced to 0.7 and 0.4, respectively.

## Discussion

We have detected a high degree of overlapping in the stress responses of *S.* Typhimurium at the transcriptional level towards heat, oxidative, acid and osmotic stresses. Such overlap could help explain the cross resistances in stress adaptation so often reported in literature [19,20]. Previous work in *Salmonella* has demonstrated that increased and cross resistance can be caused by hysteresis or memory, i.e. genes involved in resistance and induced during a stressful condition remain induced after the condition ceases [10], and a recent study in *E. coli* has demonstrated that cross-stress protection also can arise in short time due to genetic mutations [6]. Thus it may be that both memory in gene expression and short time evolution by adaptive mutations contribute to the phenomena of cross resistance.

Our network analysis revealed that the nodes degree distribution followed the power law for both transcriptional and functional (genome scale) networks. This meant that only few genes were detected as differentially transcribed in many situations and many genes were affected in few conditions, and at the genome scale few genes were involved in many reactions and most of the genes participate only in few reactions. Therefore, the highly connected nodes in these networks, the hubs, represented genes that were differentially expressed under many conditions or which had several functions in the cell. Our analysis was based on data extracted from three different strains of *Salmonella*, and we cannot rule out that details may differ between the three strains. However, the general scape of the networks should remain strain independent. Network analysis was based in the genome of *S*. Typhimurium LT2 strain, which was different from the strains used to evaluate the stress response and to carry out mutations. However, a highly similarity in the genome composition of *S*. Typhimurium strains has been previously reported [21,22]. For instance, the magnitude of the reported difference between *S*. Typhimurium strains was in one case of two genes located in prophages [21] while in another study the similarity was higher than 98% with the greatest difference attributable again to the distribution of prophages [22].

Hubs are considered the strength of scale-free networks from random failures and their Achilles’ heel for directed attacks [16]. In order to investigate whether hubs were formed by essential genes in bacterial cellular networks, we carried out directed attacks by mutation of selected hubs in both Network 2 and Network 3. This showed that deletion of genes that formed hubs in these networks did not affect growth, stress adaptation or virulence. Despite the proven essentiality of hubs in other networks, hubs do not seem to be indispensable in cellular networks. This makes cellular networks more resistant to directed attacks addressing the weakest point of the scale free topology. This conclusion was based on analyses of four out of the five most connected genes in both types of network and a limited number of stresses, and we cannot rule out that mutation affects adaptation to stresses that we have not assessed. To aid the reader in evaluation of result, a short description of our results in the light of the current knowledge of the five most connected genes in both networks is included below.

The *wraB* gene of *S.* Typhimurium encodes the WrbA protein eliciting 94% sequence similarity to the *E. coli* WrbA protein [23]. WrbA was first suggested to be involved in the binding of the tryptophan repressor to the operator [24] and recently identified as a novel flavoprotein [25] with NAD(P)H-dependent redox activity and able to reduce quinones. It has been designated as a NAD(P)H:quinone oxidoreductase (NQO) type IV which are associated with oxidative stress [26]. However, in the current investigation, a *wraB* single mutant was found not to show any changes in phenotype under any of the tested conditions, including when subjected to oxidative stress by H_2_O_2_. Investigations regarding phenotypical changes of six double mutants where *wraB* was one of the mutated genes were also performed. Four of these double mutants (*wraB/ychN, wraB/osmC, wraB/dcoC* and *wraB/cbpA*) showed a decreased ability to survive when subjected to oxidative stress by H_2_O_2_, indicating functional redundancy with these genes for oxidative stress adaptation.

In the current study, mutagenesis of *ygaU* proved unsuccessful. A comprehensive study of genes of importance for virulence in BALB/c mice has demonstrated that deletion of *ygaU* is possible, and that the gene is not essential for growth or for mouse virulence [4]. Thus, despite our difficulties, we advocate that this gene too, can be considered non-essential for growth and virulence in *S.* Typhimurium, while no results on stress adaptation are available.

*ygaU* encodes an uncharacterized protein demonstrated to be induced by salt stress in *E. coli*[27] and to be a novel member of the RpoS regulon in *S.* Typhimurium [28]. It contains a BON domain, which is characteristic of osmotic shock protection proteins [29], and a LysM domain, which was first reported in bacterial cell wall degrading enzymes and recently in other proteins with a variety of functions [30]. In the current investigation, *ygaU* was found to be significantly regulated in eight tested conditions, but due to our difficulties with construction of a defined mutant we could not assess the importance for stress adaptation.

The CbpA protein of *S.* Typhimurium elicits 89% similarity to the *E. coli* CbpA -standing for curved DNA-binding protein A- and it is induced when cells approach the stationary phase [31,32]. It is a DnaJ homolog demonstrated to act as a co-chaperone in conjunction with DnaK [33]. Regulation of CbpA activity is controlled at the transcriptional level by the RpoS and Lrp global regulators and at posttranscriptional level by degradation of CpbM by the Lon and ClpAP proteases [34]. In the current investigation, *cbpA* was significantly regulated in seven tested conditions. The *cbpA* mutant was found not to show any changes in phenotype under any of the tested conditions, and four double mutants elicited similar lack of phenotypical changes. However, three other combinations of double mutants showed significantly decreased ability to survive under H_2_O_2_ stress (*cbpA*/*wraB*, *cbpA*/*yajD* and *cbpA*/*osmC* mutants).

The UspA (universal stress protein A) superfamily is widely distributed in bacteria, *Archaea*, fungi and plants and in *E. coli* it is induced under a wide variety of stress factors [35]. The exact function of UspA is somewhat elusive, however, in some cases it appears to be of importance in defense toward DNA damaging agents and respiratory uncouplers [35]. In *S.* Typhimurium it has been demonstrated that *uspA* expression is induced during entry into stationary phase and by temperature up-shifts [36]. Furthermore, mutants have been reported to have increased sensitivity towards oxidative stress, most pronounced in the exponential growth phase, and survival in minimal media was impaired [36]. Virulence has also been reported to be affected in *uspA* mutants in both dose and infection route dependent manner [4,36]. In the current investigation, *uspA* was found to be significantly regulated in eight tested conditions. Only one double mutant, *uspA*/*siiF (STM4262),* showed a significantly decreased ability to survive when subjected to oxidative stress by H_2_O_2_.

The OsmC protein of *S.* Typhimurium shows 92% similarity to the *E. coli* OsmC identified as a member of a family of osmotically inducible proteins widely distributed in bacteria [28,37,38]. OsmC has been demonstrated to be of importance during long-term starvation of *E. coli*[39] and suggested to be a defense mechanism against oxidative stress [38]. The regulation of osmC transcription is highly complex [40,41] and it is induced when entering stationary phase and by osmotic stress or ethanol [42]. In the current investigation, *osmC* was found to be significantly regulated in seven tested conditions, but the *osmC* single mutant did not show any phenotypic change under any of the tested conditions while two of the four *osmC* double mutants, *osmC*/w*raB* and *osmC*/*cbpA*, showed a significantly decreased ability to survive when subjected to oxidative stress.

The *Salmonella* YchN protein is suggested to be a putative sulphur reduction protein. It has 92% identity to the *E. coli* YchN, but the function remains to be characterized [43]. It interacts with members of the CSD system (CsdA, CsdE and CsdL), which has been proposed to be involved in two sulphur transfer pathways: one involved in motility, while the other pathway is possibly important in stationary phase [44]. YchN was associated with 8 reactions and functions in our global genome network; despite this, the single mutant behaved like the wild type strain and we observed that only one of the double mutants deficient in *ychN* showed decreased resistance under oxidative stress.

The YajD protein is an uncharacterized protein containing a conserved HNH endonuclease signature found in viral, prokaryotic and eukaryotic proteins (NCBI domain search). The HNH superfamily includes restriction endonucleases, transposases, homing endonucleases, colicins and DNA packaging factors [45]. The gene was associated with 7 reactions and functions in the genome scale network and two double mutants in this gene showed a decreased survival under oxidative stress (Table 3).

*siiF* (*STM4262*) is present in the Salmonella Pathogenicity Island 4 (SPI-4) region [46] which is predicted to contain six genes (STM4257-4262) [47]. These genes were named *siiA-F* (*Salmonella* intestinal infection) after it was demonstrated that they were not required for systemic infection by intraperitoneal injection [17,18], but were essential for intestinal infection by oral administration [48]. However, a posterior study with intraperitoneal infection showed that some of the SPI-4 genes, although not the *siiF* gene*,* are important in long-term systemic infections in mice [49]. *STM4262* (*siiF*) has been shown to be part of a type I secretion system (*siiCDF*) encoding a putative ABC-transporter which secretes SiiE. Only one double mutant in this gene showed a decreased resistance towards oxidative stress although it is annotated with 8 reactions and functions.

The *S.* Typhimurium *dcoC* gene encodes the gamma subunit of oxaloacetate decarboxylase. The protein also contains alpha and beta subunits, and it enables anaerobic growth on citrate and tartrate [50-52]. Despite its function in central metabolism, only one double mutant showed decreased survival under H_2_O_2_ stress.

The *ybeB* gene product of *S.* Typhimurium has 97% homology to the *E. coli ybeB* gene product and homologues are widely distributed amongst bacteria and eukaryotes [53]. The *E. coli ybeB* has been shown to be associated with the large ribosomal subunit (50S) [54] and more recently, it was demonstrated to be important for survival during stationary phase as well as after transition from rich to poor medium [53]. It has been suggested that *ybeB* have a role in the down regulation of protein synthesis in stationary phase and under limited nutrition conditions by acting as a ribosomal silencing factor impairing the association of the 50S and 30S complexes. Therefore, the protein was denoted as RsfA (for ribosomal silencing factor) [53]. In our study strains with mutation in this gene were not stably obtained, which may indicate that this gene is essential.

Apart from the decreased resistance to oxidative stress, some double mutants showed attenuated virulence in mice. The apparent interactions between these genes in virulence, i.e. *wraB* with *osmC* and *cbpA* with *dcoC* is currently unknown, but the transcription of *osmC* has been shown to be upregulated 2–3 fold in murine macrophage-like J774-A.1 cells and *cbpA* to be downregulated 0.4 fold in both macrophages and HeLa cells during cell culture infections [55,56].

As discussed above, mutation of a gene forming a hub in our networks would *a priori* according to network theory have be expected to result in broad-scale phenotypical changes of the population, however; we observed that hubs seem to have redundant functionality so that single hub deletion does not impact the phenotype and viability. This could be the result of evolution since mutations with a broad scale impact would be expected to be deleterious for the cell (Fisher 1930, cited in [57]. Becker *et al.*[18] analysed 700 enzymes of *S.* Typhimurium and identified 155 enzymes that were essential for virulence. Essential enzymes were exclusively associated with a very small group of pathways specialized in the biosynthesis of products that *Salmonella* cannot efficiently obtain from its host. This agrees with our results that genes involved in a high number of functions or adaptation to environmental conditions are not essential genes.

In another study, more than 250 genes were reported to be essential for *in vitro* growth of *Salmonella* in LB-medium [58,59]. Only eight of the 257 essential genes reported in that study were detected as hubs in our genome scale network (Additional file 3: Table S3) and three in the transcriptional response to culture conditions network (Additional file 2: Table S2). These numbers support the fact that our hubs are not essential genes for growth, because a higher number of coincidences would be expected if hubs were essential genes. Two of the essential genes reported by Knuth *et al*. [58], *siiF* (STM4262) and *dcoC*, were among the genes selected for knockouts construction in our work, and contrary to their results, our analysis resulted in viable mutants. Similarly, at least another 46 of the reported essential genes in that study may actually be non-essential as independent studies demonstrated that gene inactivation resulted in viable mutants [18].

We observed that the majority of double mutations did not result in growth defects or reduced ability to adapt to stress conditions with the exception of oxidative stress*.* On the other hand, two out of five double mutants showed attenuation in mouse virulence. Many of the single non-redundant metabolic targets are already identified or too specific for *Salmonella* to be antibiotics targets [18]. A systematic approach to identify lethal double deletion using *in silico* modeling has been undertaken resulting in a list of 56 putative synthetic double deletions affecting 80 genes [59]; however the phenotype of the predicted double mutants was not experimentally assessed. Only four of those 80 genes proposed as targets for double deletions, *cysK* and *cysM*, *rfbA* and *rfbB*, were detected as hubs in our networks. Indeed, the *in silico* approach of Thiele *et al*. [59] targeted to find essential pairs of genes and hubs seem to be non-essential genes. However, the hypothesis that targeting a number of hubs could cause the disruption of the cell main functionalities sooner than if other less connected gene products are affected may lead to alternative approaches for identification of antibiotics targets.

We have seen that the number of deleted hubs required for disruption of stress resistance and virulence in *S*. Typhimurium seems to be equal to or greater than 2. Adaptive laboratory evolution experiments with *E. coli* have demonstrated a linear increase of the number of accumulated mutations as the number of generations increases, so that 45 mutations were detected after 20000 generations [60]. Assuming that the number of virulence and stress genes affected by random mutation follows a hypergeometric distribution, the probability that 2 successive random mutations affect two hubs is approximately 10^-4^ and 14 mutations, i.e. more than 6000 generations, are needed to get a value greater than 0.01 for the probability of at least two hubs are randomly mutated. This probability may be lower if considering that cellular networks can be rewired and cell behavior completely different after such a number of mutations and generations take place. Although it seems to be a small value, the real dimension of this predicted probability for cell network failure cannot be easily evaluated.

## Conclusion

In conclusion we have found that highly connected genes or hubs in cellular networks are different from essential genes. The number of deleted hubs required for the complete disruption of stress resistance and virulence in *S*. Typhimurium is 2 or more, which it may be relatively unlikely to occur spontaneously as quantified above.

## Methods

### Microarray construction

A thematic stress response and virulence microarray was constructed using Isogen Life Science platform (Maarssen, The Netherlands) by spotting 507 oligonucleotides representing 425 different genes that were predominantly related to stress and virulence onto epoxy coated glass slides (Schott Nexterion Slide E, Jena, Germany). The gene function or description used to select virulence and stress genes was derived from the *Salmonella* serovar Typhimurium LT2 genome (GenBank accession no. NC_003197) [47]. Genes were selected by selection those with genomic annotation that included one or more of the following words: stress, sigma, response, shock, stationary, osmolality, heat, cold, osmotic, decarboxylase, virulence, invasion, pathogenicity, lipopolysaccharide and antigen.

The oligonucleotides, which were designed by using Gene Runner version 3.05 and the first prototype of OligoFaktory (Delphi Genetics S.A., Charleroi-Gosselies, Belgium) [61] were synthesized and modified with a 5′-C6-amine linker by Isogen Life Science (Maarssen, The Netherlands) and spotted at a 30 mM concentration in Nexterion spotting buffer by using four Stealth AMP4 pins (ArrayIt, TeleChem International, Sunnyvale, CA) and the OmniGrid 100 spotter (Genomics Solutions, Ann Arbor, Mi.). Two hybridization areas were printed per slide and each oligonucleotide was printed twice per hybridization area. After spotting, the slides were treated for DNA immobilization, washing and blocking as recommended by the manufacturer.

### Use of published expression data

Data on regulation of the same 425 genes were extracted from published data on gene expression during the lag period and growth stages carried out with *S.* Typhimurium SL1344 [7] in addition to studies on the effect of immobilization of cells in exponential and stationary phase on gene transcription [8], and for the response to heat shock [9], all carried out with *S.* Typhimurium ST4/74 [62], which is the parental strain of the *hisG* mutant SL1344 [63].

### Hybridization conditions for transcriptional array

Gene frames for 25 μl hybridization samples (Westburg, Leusden, The Netherlands) were fit onto the hybridization areas, and covered with cleaned plastic covers (1.5×1.5 cm^2^) containing two small pierced holes and the Cy5/Cy3 labeled cDNA mixture (see below) was injected into the hybridization area. The slides were incubated for 24 hours at 42°C in a moisturized hybridization chamber. After hybridization, the Gene Frame windows were removed and the slides were incubated for 5 min in 1× SSC/0.1% SDS, next 5 min in 0.1× SSC/0.1% SDS and finally 1 min in 0.1× SSC and dried by centrifugation (440 g, 2 min).

### Analysis of hybridization results on microarray

Microarrays were scanned using the ScanArray 3000 confocal laser scanner (GSI Lumonics, Kanata, ON, Canada) by using a pixel resolution of 10 um, a Photo Multiplier Tubes value of 90% and the laserpower was set at a level observing no saturated spots. The fluorescent signals per spot and four background areas around each spot were volume measured (sVOL) by using the software package ArrayVision (Imaging Research, St. Catharines, ON, Canada). From these data the signal-to-noise ratios (S/N) were computed for each spot to discriminate true signal from noise as follows: S/N = (fluorescent spot signal - average background signal of four areas surrounding the spot)/(standard deviation of the four background area values). A commonly used threshold value to accurately quantify a signal above the noise is an S/N > 3 [64]. Prior to normalization the obtained Cy5 or Cy 3 values which had an S/N ≤ 3 were discarded. For normalization several parameters are defined: R = Cy5 value of a spot divided by the corresponding reference Cy3 spot value; H = median R value of a hybridization area calculated only from the spots that could be detected in all hybridizations; A = median H value of all hybridization areas; V = median Cy3 hybridization signal per oligo for all hybridization areas. The corrected Cy5 value per spot = R*(A/H)*V.

The fold induction/repression of gene expression under aerobic or anaerobic growth for each stress condition was calculated by dividing the mean corrected Cy5 hybridization signals (duplicate hybridizations and duplicate spots per oligonucleotide) from the stress by the non-stress sample. The fold changes of all genes being significantly differentially expressed (i) under non-stress condition in the anaerobically grown cells compared to aerobically grown cells or (ii) in the stress conditions compared to the non-stress conditions for both aerobic and anaerobic grown cells. For each gene, significantly differentially expression was tested by comparing the values of a stress condition at t = 10 min with the values of both the non-stress conditions at t = 0 and t = 10 min by using a Student t-test, P-value < 0.05 and all genes of a fold induction/repression of >1.5 were included in our comparative analysis.

### Bacterial wild type strains

*S.* Typhimurium DT104 isolate 7945, obtained from the Dutch National Institute of Public Health and the Environment (RIVM) was used to study the transcriptional response to heat, oxidative and acid stress under anoxic and oxic condition, to osmotic stress under anoxic condition and to non-stressing anoxic culture conditions by microarray hybridization. *S.* Typhimurium ST4/74 was used to construct mutants, which were used to investigate the effect of gene deletions on growth, stress adaptation and virulence. The strain was stored at -80°C in brain heart broth plus 50% glycerol (Merck, Darmstadt, Germany).

### Growth for transcriptional analysis during environmental stress

From an overnight culture of this DT104 isolate grown in brain heart broth (Merck), 0.1% was transferred to LBG pH 7.0 broth that consisted of LB broth (Difco, Detroit, Mich.) with the addition of 4 g glucose per liter and 100 mM morpholinepropanesulfonic acid (MOPS, Sigma-Aldrich, St. Louis, Mo.). Cells were cultured in LBG pH 7.0 at 37 C (referred to as non-stress condition) in three 2000 ml Erlenmeyers containing 200 ml of culture medium and shaking at 225 rpm for aerobic conditions or in fully filled 500 ml flasks without shaking for anaerobic conditions to an optical density (OD_600nm_) of around 0.30 (t = 0). Next the cultures were divided into smaller portions of 40 ml in 50 ml screw cap tubes, and subjected to several stress conditions in triplicate as explained below. Notably, the aerobic cultured cells were pooled and subsequently divided into smaller portions used in the stress treatments.

Heat stress was applied by adding 4 ml preheated LBG (+/− 82°C) to the 40 ml cultures resulting in a final temperature of 44°C. Oxidative stress was applied by adding 4 ml LBG supplemented with hydroxen-peroxide to a final concentration of 0.1 mM. Acid stress was applied by adding 4 ml LBG acidified with HCl resulting in a final pH of 5.0. Osmotic stress resulted from adding 4 ml LBG containing NaCl to give a final concentration of 1.5% in the medium. As a control, 4 ml of fresh LBG was also added to the non-stressed aerobic and anaerobic cultures. At time zero for the non-stress conditions, and after 10 min of incubation for all conditions, 40 ml culture samples were taken and added to 10 ml of an ice-cold mixture of 96% (v/v) ethanol and 5% (v/v) buffered phenol (Invitrogen, Carlsbad, CA). The tubes were centrifuged for 5 min at 1780 g at 4°C. Notably, the remaining 4 ml was used to measure the OD.

### RNA extraction and labelling for microarray hybridizations

Total RNA was isolated from the culture pellets by using TRIzol reagent (Invitrogen) and purified as described by the supplier. Notably, the TRIzol dissolved pellets of the triplicate cultures per condition were mixed. The purified RNA samples were RQ1 RNase-free DNase (Promega) treated, as described by the supplier. For each sample per hybridization, 20 μg total RNA was converted into fluorescent labelled cDNA at 37°C for two hours by using SuperScript II Reverse Transcriptase (Invitrogen) and 6 μg random hexamers (Invitrogen). Fluorescent label was directly incorporated, by using a mixture of 25 mM dATP, dGTP, dTTP, 10 mM dCTP, and 2 mM Cy3-dCTP or Cy5-dCTP (Amersham Biosciences, Piscataway, NJ). Each specific RNA sample was Cy5-dye labelled, while a mixture of all RNA samples (pooled reference) was Cy3-dye labelled. The cDNA reactions were stopped by adding 1.5 μl 20 mM pH 8.0 EDTA (Merck), subsequently treated with 0.1 M NaOH, heated for 10 min at 70°C and neutralized with 0.1 M HCl for breakdown of unconverted RNA, followed by an ethanol precipitation and dissolved in 10 μl sterile water. The sample Cy5-dye labelled cDNAs and the reference Cy3-dye labeled cDNAs were mixed (1:1) and purified for removal of uncoupled dye by using a QIAquick PCR purification kit (Qiagen, Valencia, CA), as described by the supplier. The pellets obtained were dissolved in 35 μl hybridization buffer (5x SSC, 0.2% SDS, 5x Denhardt’s solution, 50% (v/v) formamide and 0.2 ug/ul denatured herring-sperm DNA), boiled for 5 min and spun down briefly.

### Networks construction and analysis

A bipartite network, named Network 1 was constructed with the novo generated gene expression data in this study by connecting two sets of nodes: one set was formed by genes differentially transcribed under several culture conditions. The other set of nodes included the environmental conditions (heat, oxidative and acid stress in anoxic and oxic condition, osmotic stress under anoxic condition and non-stressing anoxic conditions) combined with the regulation pattern, i.e. up or down-regulation.

Network 2 was constructed by extending network with nodes representing genes and conditions to include the transcriptional response reported during the lag period, exponential growth and stationary phase [7] and in immobilized cultures in different stages [8,9].

Network 3 was a bipartite genome scale network including all genes in the genome of *S.* Typhimurium LT2 and plasmids of *S*. Typhimurium SL1344 as previously described [10]. Edges connected two sets of nodes. Genes constituted one of these sets of nodes. The genome composition was obtained from the Genome Project NCBI database [65]. The other set of nodes included metabolic pathways and cellular functions, according to the KEGG database [66], the CMR-TIGR database [67] and the COGs (Clusters of Orthologous Groups of proteins) functional categories obtained from the Genome Project NCBI database [65]. The number of nodes was 5153, from which 4717 were genes and the remaining 436 nodes represented metabolic pathways and cellular functions. There were 11626 edges between these two sets of nodes.

For networks representation and topological quantification we used the programs PAJEK [68] and Cytoscape [69]. Networks modularity was estimated implementing the fast modularity maximization algorithm [11].

### Cluster analysis

Hierarchical clustering was performed using the SAS 9.2 software [70] on the novo generated microarray data in this work using the Unweighted Pair Group Method with Arithmetic Mean (UPGMA). Expression values were coded as 1 if genes were induced, -1 if repressed and 0 if not affected. Environmental conditions (heat, oxidative and acid stress in anoxic and oxic condition, osmotic stress under anoxic condition and non-stressing anoxic conditions) were clustered according to the gene expression values.

### Construction of mutants

Cultures were grown in LB broth (Oxoid, CM1018) or on solid media consisting of LB-broth with addition of 1.5% agar. Antibiotics were used in the following concentrations: Ampicillin, 100 μg/ml; kanamycin, 50 μg/ml and chloramphenicol, 10 μg/ml. Plasmids used in DNA manipulations are listed in the Additional file 4: Table S4. Restriction enzymes and Dream-Taq polymerase (Fermentas, Thermo Scientific, Denmark) were used with the supplied buffers and according to the instruction of the manufacturer. Plasmids and PCR fragments were purified using the GeneJET Plasmid Miniprep (Thermo Scientific) - and illustra GFX PCR DNA and Gel Band Purification (GE Healthcare) kits.

Deletion mutant strains of *S*. Typhimurium 4/74 were constructed by lambda red recombination using a published protocol [71]. The pKD3 plasmid was used as the template to prevent polar effects and the primers used for generation of PCR products for the mutagenesis of the selected genes are listed in the Additional file 5: Table S5. The constructs were verified by PCR utilizing primers flanking the insertion sites, listed in in the Additional file 5: Table S5, checking for correct fragment size. Furthermore, the PCR products from the verification were sequenced (Macrogen) with the flanking primers to ensure correct constructs. Transduction into a clean wild-type background was performed with the P22 HT105/1 *int-201* phage as previously described [72] and lysogen-free colonies were obtained by streaking on green-plates followed by sensitivity testing of the colonies with the P22-H5 phage [73].

The pCP20 plasmid was used for removal of the inserted resistance gene by utilizing for FLP-FRT mediated excision [71]. The non-selective growth was performed at 37°C. Correct removal of the resistance gene was confirmed by sequencing. After removal of the resistance gene, double mutants were constructed by transduction with the previously made P22 HT105/1 *int-201* phage lysates, again ensuring lysogen-free colonies.

### Growth and stress adaptation investigations in mutants

To compare the growth ability of mutant strain to wild type strain, overnight cultures of were inoculated in LB. The strains were incubated at 37°C with shaking to balanced growth after 8–10 generations, including dilution of the cultures midway. At OD_600_ = 0.4 serial dilutions were prepared and 10 μl of the 10^-3^ to 10^-6^ dilutions were spotted on solid media of varying composition according the methods described elsewhere [74]: 1) Standard LB plates were incubated at different temperatures; 15°C, 37°C and 44°C; 2) growth at different NaCl concentrations were examined by spotting onto plates supplemented to contain an additional 2% or 4% NaCl; 3) growth at different pH values was investigated by plating onto plates where the pH values were: 5, 9, 10 and 11. These plates were prepared by mixing filter sterilized liquid LB medium at high or low pH with normal autoclaved LB media at predetermined ratios. The autoclaved LB media was supplemented with agar to obtain a final concentration of 1.5%.

H_2_O_2_ killing-assays were performed by adjusting the optical density of overnight cultures of wild type and mutant, followed by mixing in a 1:1 ratio. The mixture was used for inoculation of LB (OD_600_ = 0.02) that was incubated at 37°C with shaking. At OD_600_ = 0.4 a sample was taken for determination of bacterial count and determination of wild type to mutant ratio prior addition of H_2_O_2_ to a final concentration of 15 mM. The culture was again sampled for bacterial count and the ratio determination after incubation for an additional 30 min. The wild type to mutant ratio was determined by plating onto plates with or without chloramphenicol.

### Virulence of mutants in mice

The optical density of overnight cultures of wild type and mutant in LB were adjusted and the cultures mixed in a 1:1 ratio. Groups of 5 C57BL/6 mice were infected with 100 μl of diluted bacterial culture by intra-peritoneal (i.p.) challenge at a total final dose of 10^4^ bacteria. The infection was allowed to proceed up to 6 days, unless the animals were clearly affected, in which case they were humanely killed. Euthanization was performed by cervical dislocation followed by removal and homogenization of the spleen. Serial dilutions of the homogenate as well as of the initial mixed culture used for inoculation were made and plated onto LB plates. Following the incubation of the plates at 37°C, the ratio of mutant to wild type was determined by randomly picking 100 colonies that were transferred to LB plates with or without chloramphenicol as previously described [75]. The competitive index was calculated as the mutant/wt ratio in the spleen versus the mutant/wt ratio of the inoculum. Experiments were conducted with permission to John Elmerdahl Olsen from the Danish Animal Experiments Inspectorate, license number 2009/561-1675.

### Statistical analysis

Comparison of competitive indexes based on bacteria obtained from spleen of mice and CFU of bacteria was done by paired T-test.

### Accession numbers

The array design and the microarray datasets have been deposited with ArrayExpress database (accession numbers: A-MEXP-2343 and E-MTAB-1804, respectively).

## Competing interests

The authors declare they have no competing interests.

## Authors’ contributions

CP, HA, LET and JEO planned the study, CP performed network analysis, JTR and HA performed experimentation, MR, GK, MBN, HA, TA and MZ provided datasets for analyses, JEO, JTR and CP drafted the manuscript and all authors approved of the final manuscript.

## Supplementary Material

Additional file 1: Table S1 Ratio values between the intensities of two conditions as depicted below exhibiting a significant (P < 0.05) change between both conditions. Click here for file

Additional file 2: Table S2 Hubs or highly connected genes to culture conditions in the transcriptional network of *S.*Typhimurium, i.e. genes differentially transcribed under heat, oxidative, acid and/or osmotic stress and/or anaerobic condition, lag phase, exponential growth, stationary phase and immobilization. Click here for file

Additional file 3: Table S3 Hubs or highly connected genes to cellular functions and metabolic pathways in the genome scale network for *S.* Typhimurium Click here for file

Additional file 4: Table S4 Plasmids and Phages used in DNA manipulations. Click here for file

Additional file 5: Table S5 Sequnce of primers used in the study. Click here for file
