# Supplementary material for: Non-essential genes form the hubs of genome scale protein function and environmental gene expression networks in Salmonella enterica serovar Typhimurium
Source: BMC Microbiol. 2013 Dec 17;13:294. doi: 10.1186/1471-2180-13-294 (PMC3878590; doi:10.1186/1471-2180-13-294)
Supplement: Additional file 1: Table S1 — Ratio values between the intensities of two conditions as depicted below exhibiting a significant (P < 0.05) change between both conditions. [file 1471-2180-13-294-S1.pdf]

Table S1. Ratio values between the intensities of two conditions as depicted below exhibiting a significant (P<0.05) change between both conditions

Nucleotides position in chromosome (LT2)	Locus Tag (LT2)	Gene	Gene function/description	Stress/No stress for:							No O_2 /O_2
				Heat		H_2O_2		Acid		NaCl	No stress
				No O_2	O_2	No O_2	O_2	No O_2	O_2	No O_2	
3167872..3170031	STM3010	*aas*	bifunctional: 2-acylglycerophospho-ethanolamine acyl transferase; acyl-acyl carrier protein synthetase		0.8		0.7	0.7			
178918..180807	STM0153	*aceF*	pyruvate dehydrogenase, dihydrolipoyltransacetylase component			2.4					0.4
2363134..2364195	STM2265	*ada*	bifunctional: O6-methylguanine-DNA methyltransferase; transcription activator/repressor (AraC/Xyl family)				1.9				
1843128..1845806	STM1749	*adhE*	alcohol dehydrogenase / acetaldehyde dehydrogenase		0.6	0.1	2.4	0.3	0.4	0.1	6.8
4538697..4540967	STM4296	*adi*	*adiA* , arginine decarboxylase, catabolic; inducible by acid		1.8	10.3		45.1	2.3		
4619596..4621236	STM4377	*aidB*	acyl-CoA dehydrogenase, adaptive response (transcription activated by Ada)		2.1			0.7			
714545..716473	STM0652	*atoC*	putative sigma 54 dependent transcriptional regulator (=STM0652)	1.8	1.5	5	3.5	1.9	1.6		
2472873..2474300	STM2361	*atoS*	sensor protein AtoS for response regulator atoC (=STM2361)	1.2		0.6		0.4	0.7	0.2	1.6
4075520..4076383	STM3866	*atpG*	membrane-bound ATP synthase, F1 sector, gamma-subunit	1.8	0.6	0.6	0.3	0.5	0.5		0.6
3009898..3010806	STM2865	*avrA*	putative inner membrane protein	2.1	2.5	28	7.2	8.7	2.3		
501001..501318	STM0446	*bolA*	morphogene, involved in modulating cell morphology, putative regulator of murein genes		2.8		5.1		1.5	157.0	0.3
2699950..2702094	STM2559	*cadA*	lysine decarboxylase	1	1.7	1.1	1.7	3.5		0.2	1.3
2698536..2699867	STM2558	*cadB*	lysine/cadaverine transport protein					2.6	13.7		
75880..77028	STM0066	*carA*	carbamoyl-phosphate synthetase, glutamine-hydrolysing small subunit, regulated by arginine	1.3	0.4	0.1	0.1	0.1	0.1	0.1	0.7
77047..80274	STM0067	*carB*	carbamoyl-phosphate synthase, large subunit, regulated by arginine	1.9	0.4	0.2			0.2		
1198999..1199919	STM1112	*cbpA*	curved DNA-binding protein	1		1.7		1.6	1.4		1.7
260126..260983	STM0222	*cdsA*	CDP-diglyceride synthase, sigma E regulon transcribed as a three gene operon			0.3	0.5	0.7	0.5		
2014756..2016417	STM1919	*cheM*	methyl accepting chemotaxis protein II, aspartate sensor-receptor		0.8	1.4	2.5	0.3			
2802116..2804689	STM2660	*clpB*	ATP-dependent protease, Hsp 100, part of novel multi-chaperone system with DnaK, DnaJ, and GrpE	3	1.3	2.6	2.8	5		5.7	2.2
503210..503833	STM0448	*clpP*	specificity component of ATP-dependent serine protease with clpX, degradates RpoS when bound to MviA	1.2		2.4	1	1.5			
1306775..1307596	STM1221	*cobB*	putative nicotinate-nucleotide dimethylbenzimidazo-lephosphoribosltransferase, homolog of virulence factor	0.8	0.6						
4270595..4271095	STM4060	*cpxP*	periplasmic repressor of cpx regulon by interaction with CpxA, rescue from transitory stresses		3.5		1.2	18.1	10.9		0.9
3615645..3616277	STM3466	*crp*	catabolite activator protein (CAP), cyclic AMP receptor protein (CRP family)	0.7	0.6	0.3	0.7	0.6	1.3		1.5
1231133..1231588	STM1143	*csgB*	minor curlin subunit precursor, nucleator for assembly of adhesive surface organelles		2.8						
3836439..3836651	STM3649	*cspA*	major cold shock protein 7.4, transcriptional activator of hns	0.4	0.5	0.6	0.2		0.5		0
897487..897990	STM0831	dps	DNA starvation/stationary phase protectio							++	

3203688..3204401	STM3043	*dsbC*	protein disulfide isomerase II, acting on folding of enveloppe proteins						1.3		
2068650..2068739	STM1985	*dsrA*	a small RNA antisilencer of the H-HS-silenced rdsA gene			++	10.2				
2068089..2068296	STM1983	*dsrB*	regulatory RNA, regulated by DsrA and HNS		1.4	10.5	4	3.4	1.9	11	0.6
2961778..2962950	STM2814	*emrA*	multidrug resistance secretion protein, stationary phase	0.8	0.7	2				0.2	0.7
2962967..2964505	STM2815	*emrB*	putative MFS superfamily, multidrug transport protein, stationary phase	0.8	0.6	0.6	0.7	1.4			
640808..641512	STM0584	*entD*	enterochelin synthetase, component D			1.9	2		0.6		
4188137..4189300	STM3982	*fadA*	3-ketoacyl-CoA thiolase; (thiolase I, acetyl-CoA transferase), in complex with FadB catalyzes			2.2	3.8				
4189310..4191499	STM3983	*fadB*	3-hydroxyacyl-coA dehydrogenase			3.3			0.8		
2502389..2503702	STM2391	*fadL*	transport of long-chain fatty acids; sensitivity to phage T2, putative binding site for ompR	0.6	0.4	0.4			0.1		0.3
1905889..1906608	STM1805	*fadR*	negative regulator for fad regulon and positive activator of fabA (GntR family)			2.4					
223738..225927	STM0191	*fhuA*	outer membrane protein receptor / transporter for ferrichrome, colicin M, and phages T1, T5, and phi80			8.5			0.04		0.1
3620354..3620956	STM3470	*fic*	cell filamentation protein, stationary phase induced gene, affects cell division						1.5		
3556262..3556558	STM3385	*fis*	site-specific DNA inversion stimulation factor, represses rpoS expression	1	0.6	0.03	0.2	0.1	0.2	0.1	0.5
4633932..4634552	STM4397	*fklB*	FKBP-type 22KD peptidyl-prolyl cis-trans isomerase (rotamase)	0.6	0.3	0.1	0.4	0.4	0.5		
3604871..3605689	STM3453	*fkpA*	FKBP-type peptidyl-prolyl cis-trans isomerase (rotamase), acting on folding of enveloppe proteins	0.6	0.4	0.3	0.6	4.3	3.6		
757128..757658	STM0694	*fldA*	flavodoxin 1, putative binding site for fur		0.9	0.3		0.8			0.8
1257046..1257339	STM1172	*flgM*	anti-FliA (anti-sigma) factor; also known as RflB protein		0.9	2.7	3.5	1.1	2.3		
2021717..2022067	STM1925	*flhD*	regulator of flagellar biosynthesis, acts on class 2 operons, putative binding site for ompR	1.6	1.6	0.2	0.7	0.8	0.5		1.4
2047658..2049145	STM1959	*fliC*	flagellar biosynthesis, flagellin, filament structural protein, phoPQ represses transcription of fliC	1.1	1.1	3.3	1.6	1			
100839..101318	STM0087	*folA*	dihydrofolate reductase type I, trimethoprim resistance	0.7	0.9	0.2		1.5		0.2	0.8
2290444..2291112	STM2193	*folE*	GTP cyclohydrolase I		0.6	5	1.4		0.9		0.5
154360..155622	STM0132	*ftsA*	ATP-binding cell division protein, septation process, complexes with FtsZ, junctions of inner and outer membranes	1.5			1.3		0.8		
1037371..1041426	STM0960	*ftsK*	cell division protein, required for cell division and chromosome partitioning, regulates UspA	0.9	0.6			0.6	0.8		
4301113..4302087	STM4093	*ftsN*	essential cell division protein		0.6	0.6					
153533..154363	STM0131	*ftsQ*	cell division protein; ingrowth of wall at septum		0.9	0.7	0.6	0.6	0.7		0.6
155683..156834	STM0133	*ftsZ*	tubulin-like GTP-binding protein and GTPase, forms circumferential ring in cell division		0.9		0.9				
756394..756846	STM0693	*fur*	major iron regulator	0.9	0.9	3.5	1.8		0.8		0.4
2943010..2944410	STM2793	*gabP*	APC family, gamma-aminobutyrate transport protein	1.3		1.8					
838373..839521	STM0774	*galK*	galactokinase		0.9	1.1	1			0.2	1.4
1368693..1369688	STM1290	*gapA*	glyceraldehyde-3-phosphate dehydrogenase A		0.4	0.4	0.6	2.5	0.9		1.9
3706095..3708281	STM3538	*glgB*	1,4-alpha-glucan branching enzyme	1.5	1.5		1.7	1.4	2.7		
3702812..3704107	STM3536	*glgC*	glucose-1-phosphate adenylyltransferase		1.3		2.8		1.4		1.4

3359314..3359523	STM3197	*glgS*	glycogen biosynthesis, rpoS dependent		2.1	6.3		1.2			
3933098..3933721	STM3740	*gmk*	guanylate kinase		0.7		0.8		0.9		0.6
2821587..2822177	STM2681	*grpE*	molecular chaparone, heat shock protein	2	1.1	0.6	1	3.4			2.2
1250351..1250998	STM1165	*grxB*	glutaredoxin 2		1.3		1.9		1.7		1.3
1525740..1526345	STM1451	*gst*	glutathionine S-transferase			1.8	1.7	0.6			1.2
3012107..3012994	STM2867	*hilC*	invasion regulatory protein							4.1	
2678197..2680047	STM2539	*hscA*	chaperone, member of Hsp70 protein family, believed to be involved in assembly of Fe-S clusters	0.6	0.3	0.5			0.6	0.2	0.9
722533..724212	STM0659	*hscC*	putative heat shock protein, homolog of hsp70 in Hsc66 subfamily	1.4	2.4						1.3
4299150..4300481	STM4091	*hslU*	ATPase component of the HslUV protease, rpoH controlled heat shock response	1		0.4		4.5			1.8
4300491..4301021	STM4092	*hslV*	peptidase component of the HslUV protease, rpoH controlled heat shock response	1.2				4.6	0.2		1.4
10092..10805	STM0010	*htgA*	positive regulator for sigma H (sigma 32) promoters, permitting growth at high temperature		1.1	4.5	5.3	2.2	2.6		
543926..545800	STM0487.S	*htpG*	chaperone Hsp90, heat shock protein C	4.3	1.3	0.8	1	4.8		6.1	6.5
244492..245919	STM0209	*htrA*	periplasmic serine protease Do, heat shock protein, transcribed by rpoE	1.3	1.7		0.5	10.1	7.9		1.4
2999127..2999588	STM2853	*hycA*	transcriptional repressor of hyc and hyp operons	0.2				3.3			
2998373..2998981	STM2852	*hycB*	hydrogenase-3, iron-sulfur subunit (part of FHL complex)	0.5	0.6	0.3					4.4
2995621..2996544	STM2850	*hycD*	hydrogenase 3, membrane subunit (part of FHL complex)					0.9			
2993342..2993884	STM2848	*hycF*	hydrogenase 3, putative quinone oxidoreductase	0.3		0.4		1.9			7.3
2992575..2993342	STM2847	*hycG*	hydrogenase activity		1.3						
2992168..2992578	STM2846	*hycH*	processing of HycE (part of the FHL complex)	0.4		0.6		1.5		0.1	4.7
2999798..3000154	STM2854	*hypA*	guanine-nucleotide binding protein in formate-hydrogenlyase system, nickel donor for HycE of hydrogenlyase 3	0.5		0.6		1.6			
3000223..3001095	STM2855	*hypB*	hydrogenase-3 accessory protein, assembly of metallocenter						2.1		2.4
3001086..3001358	STM2856	*hypC*	putative hydrogenase expression/formation protein			0.3			2.4		
3001358..3002476	STM2857	*hypD*	putative hydrogenase expression/formation protein						1.7		
3002473..3003483	STM2858	*hypE*	putative hydrogenase expression/formation protein		0.9	0.5		0.7			2
3312644..3313762	STM3150	*hypO*	putative Ni/Fe hydrogenases, small subunit		1.9	0.1		0.2		0.01	11.4
3024412..3024660	STM2881	*iacP*	putative acyl carrier protein, invasion	1.8	3.3		0.3		0.5		0.3
3021535..3022017	STM2877	*iagB*	cell invasion protein	1.1	3.5						1.4
3038400..3040457	STM2896	*invA*	invasion protein	0.6				0.7			
3037969..3038376	STM2895	*invB*	surface presentation of antigens; secretory proteins		1.7		0.7	0.9	0.5		2.5
3036677..3037972	STM2894	*invC*	surface presentation of antigens; secretory proteins	0.6	1.6						2.1
3043282..3043932	STM2899	*invF*	invasion protein, regulates SPI-1			1.6			0.7		0.9
3041597..3043285	STM2898	*invG*	invasion protein; outer membrane						0.6		
3044389..3044832	STM2900	*invH*	invasion protein	0.7	0.8		0.4		0.7		
3036256..3036699	STM2893	*invI*	surface presentation of antigens; secretory proteins						0.5		
3035246..3036256	STM2892	*invJ*	surface presentation of antigens; secretory proteins	0.7	1.5						2
1397114..1399366	STM1318	*katE*	catalase, hydroperoxidase HPII(III)	1.8	1.2	3.9	3	2.8	1.6	144.2	
4319583..4321763	STM4106	*katG*	catalase, hydroperoxidase HPI(I)			184.8	170.4				1.6
3919338..3920615	STM3724	*kdtA*	3-deoxy-D-manno-octulosonic-acid transferase (KDO transferase)		1.9	2.7		0.6			1.4

3920624..3921103	STM3725	*kdtB*	phosphopantetheine adenylyltransferase		0.6					0.2	
505542..507896	STM0450	*lon*	DNA-binding, ATP-dependent protease la; cleaves RcsA and SulA, heat shock k-protein (DNA binding activity)					1.5			1.7
266995..267783	STM0228	*lpxA*	UDP-N-acetylglucosamine acetyltransferase, lipid biogenesis	0.9	1.1	0.5	0.7	0.4	0.6		
4169762..4172026	STM3965	*metE*	5-methyltetrahydropteroyltriglutamate-homocysteine S-methyltransferase			1.8					
4699688..4702396	STM4456	*mgtA*	P-type ATPase, Mg2+ ATPase transporter	1.3	6.5			0.6			
3961542..3964268	STM3763	*mgtB*	Mg2+ transport protein	1.1	17.9	1.3		1.7	6.8		
844442..845230	STM0779	*modE*	transcriptional repressor of modABCD operon (molybdate uptake)			1.2					
4575111..4576757	STM4330	*mopA*	chaperone Hsp60 with peptide-dependent ATPase activity, affects cell division	4.6	1.8		1	2.8	1		5.8
4574774..4575067	STM4329	*mopB*	chaperone Hsp10, affects cell division	4.8	2.2			3			5
3543516..3544559	STM3374	*mreB*	rod shape-determining protein; HSP70 class molecular chaperones involved in cell morphogenesis		0.8	0.2	0.4	0.6	0.6		
1329466..1329705	STM1241	*msgA*	Macrophage survival gene; reduced mouse virulence	1.6	2.2	4.7	3.7				
3877404..3878552	STM3686	*mtlD*	mannitol-1-phosphate dehydrogenase	1.2	1.5			1.2			1.8
1080209..1084675	STM0994	*mukB*	kinesin-line cell division protein involved in sister chromosome partitioning		0.6	0.5	0.4	0.4	0.5		
3050279..3052846	STM2909	*mutS*	methyl-directed mismatch repair, recognize exocyclic adducts of guanosine		1.2	2.4					
1253776..1254699	STM1169	*mviM*	putative virulence factor		0.5				0.7		
1254964..1256538	STM1170	*mviN*	putative virulence factor	1.5		0.7	0.8		0.8		
1660688..1664428	STM1577	*narZ*	nitrate reductase alpha chain						1.5		
637028..637681	STM0578	*nfnB*	dihydropteridine reductase/oxygen-insensitive NAD(P)H nitroreductase	0.8	0.5						1.4
2602992..2604026	STM2488	*nlpB*	lipoprotein-34	0.7	0.6	0.2	0.5	0.4	0.8		
3066549..3067682	STM2925	*nlpD*	lipoprotein (upsteam of rpoS)			10.2	2	2.9	1.5	37.4	0.2
2332130..2333959	STM2232	*oafA*	O-antigen five: acetylation of the O-antigen (LPS)			0.1		0.2	0.3		0.4
3384678..3386084	STM3218	*oat*	putative acetylornithine aminotransferase				2.7		2		
1753663..1754178	STM1659	*ogt*	O-6-alkylguanine-DNA/cysteine-protein methyltransferase, stationary phase		1.4	4.2	2	7.8	3		
2365443..2366579	STM2267	*ompC*	outer membrane protein 1b (ib;c), porin		0.4		0.7	0.1	0.2	0.2	
1089781..1090872	STM0999	*ompF*	outer membrane protein 1a (ia;b;f), porin	0.8	0.8	1.3	0.7	1.6	2.6		
3659606..3660325	STM3502	*ompR*	stationary phase transcription response regulator in two-component regulatory system with EnvZ		1	2.4	0.8		0.5		0.1
1461740..1463083	STM1379	*orf48*	putative amino acid permease					2.8			
	PSLT012	*orf7*	putative bacterial regulatory proteins, luxR family		3.8						
1473820..1474032	STM1388	*orf70*	putative cytoplasmic protein	0.8			0.8	0.6	0.6		0.4
3013788..3014468	STM2869	*orgA*	putative flagellar biosynthesis/type III secretory pathway protein		0.9	0.2	0.4	1.7			0.6
1799923..1800141	STM1705	*osmB*	osmotically inducible lipoprotein			2	3.4	4.9	6	4.4	0.8
1644237..1644668	STM1563	*osmC*	resistance protein, osmotically inducible		1.5	2.8	3.6	2.1	1.6	18.5	
4815879..4816496	STM4561	*osmY*	hyperosmotically inducible periplasmic protein, stationary phase	1.5	2.5	16.8	6.1	7.1	5.1	++	0.5
2023300..2024721	STM1928	*otsA*	trehalose-6-phosphate synthase, stationary phase	1.4	1.3	3.4	2.6	2.4	2		
2024696..2025499	STM1929	*otsB*	trehalose-6-phosphate phophatase, biosynthetic, stationary phase		2.4	6.4	6.1	3.4	3.4	227.5	
1175548..1176228	STM1087	*pipA*	Pathogenicity island encoded protein: SPI3			1.1		2.9			

1176450..1177325	STM1088	*pipB*	Pathogenicity island encoded protein: SPI3	1.8	6.7	4.2	1.5	9.4	8	++	
1177873..1178214	STM1090	*pipC*	Pathogenicity island encoded protein: homologous to ipgE of Shigella	2	3.1	2.3		1.2			
1180638..1182107	STM1094	*pipD*	Pathogenicity island encoded protein: SPI3		1.6		2.3				
4532639..4533709	STM4291	*pmrA (basR)*	response regulator in two-component regulatory system with BasS (OmpR family)	0.3	0.4	0.6	0.6	1.7	3.1		0.6
4533719..4534387	STM4292	*pmrB (basS)*	sensory kinase in two-component regulatory system with BasR	0.4	0.3	1.4	0.6	2.4			0.5
2411318..2411575	STM2304	*pmrD*	polymyxin resistance protein B		1.5	2.1	2.8	2.4	5.6		
2405113..2406096	STM2298	*pmrF*	putative glycosyl transferase, PhoPQ regulated via PmrAB	0.3	0.2		0.5	5.8	3.6		
4206131..4208917	STM3999	*polA*	DNA polymerase I			1.6	1.6	0.6	0.9		
762063..763382	STM0700	*potE*	APC family, putrescine/ornithine antiporter	0.3		0.5		0.5			
3048771..3049427	STM2907	*pphB*	serine/threonine specific protein phosphatase 2		1.5	1.5	0.6		0.8		0.5
3621222..3621794	STM3472	*ppiA*	peptidyl-prolyl cis-trans isomerase A (rotamase A)			0.4	0.6	2.1			0.7
598468..598962	STM0536	*ppiB*	peptidyl-prolyl cis-trans isomerase B (rotamase B)	1	0.8	1.2		1.2	0.9		0.8
4118944..4119225	STM3910	*ppiC*	peptidyl-prolyl cis-trans isomerase C (rotamase C)	0.6	0.7	0.5			0.6		0.4
1619783..1621339	STM1544	*pqaA*	PhoPQ-regulated protein	1.1	1.5			8	9		0.8
3016342..3017520	STM2874	*prgH*	cell invasion protein	0.4					0.9		
3016075..3016317	STM2873	*prgI*	cell invasion protein; cytoplasmic	0.7	0.9			0.5	0.6		2.1
3015751..3016056	STM2872	*prgJ*	cell invasion protein; cytoplasmic					0.7			2.1
3014996..3015754	STM2871	*prgK*	cell invasion protein; lipoprotein, may link inner and outer membranes	0.6			0.9	0.6	0.7		2.2
4530970..4532472	STM4290	*proP*	MFS family, low-affinity proline transporter (proline permease II)	1.4	3	3.1	1.3	2.7	2.2	++	
2955951..2957153	STM2809	*proV*	ABC superfamily (atp_bind), glycine/betaine/proline transport protein, stationary phase	1.3			0.3	0.5		++	0.6
2957146..2958210	STM2810	*proW*	ABC superfamily (membrane), glycine/betaine/proline transport protein, stationary phase			1.4					
2958280..2959275	STM2811	*proX*	ABC superfamily (bind_prot), glycine/betaine/proline transport protein, stationary phase				0.3			++	0.4
4609096..4610394	STM4366	*purA*	adenylosuccinate synthetase		0.6	0.5		0.6	0.8	0.2	
4391161..4392450	STM4175	*purD*	phosphoribosylglycinamide synthetase (GAR synthetase)	0.8	1	0.5	0.6	0.7	0.6		0.7
639016..639357	STM0581	*ramA*	putative regulatory protein, resistance against oxidative stress				0.8				
	PSLT009	*rcK*	resistance to complement killing	2.2		2			0.7		
2067427..2068050	STM1982	*rcsA*	positive transcriptional regulator of capsular/exo-polysaccharide synthesis (LuxR/UhpA family)	0.7	1.7	1.2	1.7	2.8	7.3		0.6
2369994..2370644	STM2270	*rcsB*	response regulator (positive) in two-component regulatory system with RcsC (LuxR/UhpA familiy)	0.8	0.4			1.3			
2370747..2373593	STM2271	*rcsC*	sensory histidine kinase in two-component regulatory system with RcsB, regulates colanic capsule biosynthesis	0.6				0.6			
3144276..3146111	STM2993	*recD*	exonuclease V, alpha chain		0.8						
3102880..3105114	STM2956	*relA*	ppGpp synthetase I (GTP pyrophosphokinase), ppGpp act as positive signal for rpoS transcription		1.3	1.1			1.6		1.2
3914534..3915613	STM3719	*rfaB*	UDP-D-galactose:(glucosyl)lipopolysaccharide-1,6-D-galactosyltransferase	0.8	0.5						1.3
3907297..3908250	STM3712	*rfaC*	heptosyl transferase I	0.8	0.4	0.2		0.4	0.6	0.2	

3910807..3911616	STM3715	*rfaZ*	lipopolysaccharide core biosynthesis		0.7		0.6				0.5
2175670..2176548	STM2095	*rfbA*	dTDP-glucose pyrophosphorylase	0.6	0.6	0.3	0.4	0.6	0.4	0.1	0.5
2177495..2178580	STM2097	*rfbB*	dTDP-glucose 4,6 dehydratase	0.8	0.6	0.2	0.5	0.4	0.5	0.2	0.6
2175118..2175669	STM2094	*rfbC*	dTDP-4,deoxyrhamnose 3,5 epimerase	0.7	0.8	0.3	0.4	0.3	0.5	0.2	0.6
2176596..2177495	STM2096	*rfbD*	TDP-rhamnose synthetase	0.7	0.5	0.4	0.4	0.4	0.5	0.2	0.7
2173350..2174123	STM2092	*rfbF*	LPS side chain defect: glucose-1-phosphate cytidylyltransferase	0.5	0.6	0.5	0.3	0.4	0.4	0.1	0.6
2172266..2173345	STM2091	*rfbG*	LPS side chain defect: CDP glucose 4,6-dehydratase	0.7	0.7	0.5	0.4	0.3	0.5	0.2	0.7
2170926..2172239	STM2090	*rfbH*	LPS side chain defect: CDP-6deoxy-D-xylo-4-hexulose-3-dehydrase	0.7	0.8	0.7	0.3	0.6	0.6	0.2	
617360..617722	STM0559	*rfbI*	LPS side chain defect: CDP-6-deoxy-delta3,4-glucoseen reductase	0.8	0.8	0.2	0.3	0.3	0.4	0.2	0.6
2169999..2170898	STM2089	*rfbJ*	LPS side chain defect: CDP-abequose synthase	0.7		0.6	0.4		0.6	0.2	
2162433..2163866	STM2083	*rfbK*	LPS side chain defect: phosphomannomutase	0.8	0.7	1.6	0.9	0.7	0.8		0.6
2163853..2165292	STM2084	*rfbM*	LPS side chain defect: mannose-1-phosphate guanylyltransferase	0.7	0.6	0.7	0.5		0.6		0.5
2165293..2166237	STM2085	*rfbN*	LPS side chain defect: rhamnosyl transferase	0.8	0.8		0.6	0.6	0.7		0.4
2160931..2162361	STM2082	*rfbP*	LPS side chain defect: bifunctional enzyme, undecaprenol-phosphate galactosephosphotransferase/O-antigen transfer	0.7	0.6	0.6	0.6	0.7	0.6		0.5
2166238..2167299	STM2086	*rfbU*	LPS side chain defect: mannosyl transferase		0.9	1.4	0.7	0.7	0.7		0.4
2167619..2168620	STM2087	*rfbV*	LPS side chain defect: abequosyltransferase	0.9	1	0.8	0.7		0.9		0.4
2168625..2169917	STM2088	*rfbX*	LPS side chain defect: putative O-antigen transferase			0.8	0.8	0.7	0.8		0.4
3583301..3584290	STM3415	*rpoA*	DNA-directed RNA polymerase subunit alpha	0.9	1.1	0.5	0.3	0.1	0.3	0.2	0.8
4365908..4369936	STM4153	*rpoB*	RNA polymerase, beta subunit	0.9	0.7	0.4	0.4	0.3	0.4		
4370013..4374236	STM4154	*rpoC*	RNA polymerase, beta prime subunit	0.7	0.8	0.5	0.3	0.3	0.7	0.2	
3376401..3378248	STM3211.S	*rpoD*	sigma D (sigma 70) factor of RNA polymerase, major sigma factor during exponential growth		0.4	0.7		5.8			
2779428..2780003	STM2640	*rpoE*	sigma E (sigma 24) factor of RNA polymerase, response to periplasmic stress, also important in stationary phase	0.9	0.9		1.6	5.5	2.8		1.2
2044747..2045466	STM1956	*rpoF(fliA)*	sigma F (sigma 28) factor of RNA polymerase		0.3	1.8		0.4	0.7		2.2
3735927..3736781	STM3568	*rpoH*	sigma H (sigma 32) factor of RNA polymerase	1.7	1.8	1.5	1.5	2.4	2.3		1.4
3484410..3485843	STM3320	*rpoN*	sigma N (sigma 54) factor of RNA polymerase		0.4	0.4	0.6	1.4			0.6
3065494..3066486	STM2924	*rpoS*	sigma S (sigma 38) factor of RNA polymerase, major sigmafactor during stationary phase	1.4	1.3	4	1.5	1.3	1.2	10.6	0.4
3933776..3934051	STM3741	*rpoZ*	RNA polymerase, omega subunit		0.4	0.6	0.6	0.7	0.7		
4382750..4383238	STM4165	*rsD*	regulator of sigma D, has binding activity to the major sigma subunit of RNAP		1		6.1		2.1		
2778746..2779396	STM2639	*rseA*	anti sigma E (sigma 24) factor, negative regulator	0.9	0.7			8	4.6		1.2
2777790..2778746	STM2638	*rseB*	anti sigma E (sigma 24) factor, negative regulator			1.5		3	1.7		1.6
2777314..2777793	STM2637	*rseC*	regulator of sigma E (sigma 24) factor	0.8	0.8		1.6	1.7	1.5		0.7
3684868..3686451	STM3522	*rtcR*	sigma N (sigma 54)-dependent regulator of rtcBA expression (EBP familiy)	2.3		4.5	++	2.4	1.4		
2039658..2040380	STM1950	*sdiA*	transcriptional regulator of ftsQAZ gene cluster (LuxR/UhpA family), regulator of rck operon on virulence plasmid		0.5	5.6	2.3	0.5			0.6
3030898..3031395	STM2886	*sicA*	surface presentation of antigens; secretory proteins		1.3	3.1					1.8
3023689..3024081	STM2879	*sicP*	chaparone, related to virulence on SPI, acts as a specific chaperone for SptP	1.8	3.6	1.2	0.3		0.5	93.9	0.3

3024679..3026736	STM2882	*sipA*	cell invasion protein		1.8				0.7	2.2
3029114..3030895	STM2885	*sipB*	cell invasion protein	1.6	1.2	1.9		0.6	0.6	2.5
3027857..3029086	STM2884	*sipC*	cell invasion protein		1.5	1.7	1.3		0.9	4.4
3026755..3027786	STM2883	*sipD*	cell invasion protein		1.6		0.8	0.6	0.7	3
3006368..3007285	STM2861	*sitA*	Salmonella iron transporter: fur regulated		0.2	310.1	31.3		0.5	
3034335..3035246	STM2891	*spaO*	surface presentation of antigens; secretory proteins		1.8					
3033671..3034345	STM2890	*spaP*	surface presentation of antigens; secretory proteins	0.7	3.1				0.6	1.7
3032590..3033381	STM2888	*spaR*	surface presentation of antigens; secretory proteins				0.5		0.6	
763379..765577	STM0701	*speF*	ornithine decarboxylase isozyme, inducible	0.5	2.1		1.5	4.2	1.2	2.7
3934070..3936181	STM3742	*spoT*	bifunctional : ppGpp synthetase II; also guanosine-3',5'-bis pyrophosphate 3'-pyrophosphohydrolase		0.4	0.2	0.4	0.5	0.4	
3010967..3011722	STM2866	*sprB*	transcriptional regulator		2.9	1.3	1.8	4.6	1.7	1.8
3022071..3023702	STM2878	*sptP*	protein tyrosine phosphate	2.3	2.2		0.3	0.7	0.4	0.4
	PSLT040	*spvA*	Salmonella plasmid virulence: outer membrane protein			4.6				0.1
	PSLT039	*spvB*	Salmonella plasmid virulence: hydrophilic protein			2.9				
	PSLT037	*spvD*	Salmonella plasmid virulence: hydrophilic protein	1	0.6	1.7				
	PSLT041	*spvR*	Salmonella plasmid virulence: regulation of spv operon, lysR family		12.1	2				
	PSLT010	*srgB*	sdiA-regulated gene; putative outer membrane protein		1.6					
	PSLT007	*srgC*	sdiA-regulated gene;putative bacterial regulatory helix-turn-helix proteins, araC family	1.6	4	2.7	1.7	1.3	1.8	
1479999..1480400	STM1393	*ssaB*	Secretion system apparatus	0.7					0.7	
1480402..1481895	STM1394	*ssaC*	Secretion system apparatus			7.6				
1481876..1483087	STM1395	*ssaD*	Secretion system apparatus	0.6						
1483095..1483337	STM1396	*ssaE*	Secretion system effector		3.4					
1489537..1489752	STM1406	*ssaG*	Secretion system apparatus			1.7				
1491589..1492263	STM1411	*ssaK*	Secretion system apparatus			1.6				
1492229..1493245	STM1412	*ssaL*	Secretion system apparatus						0.7	
1497353..1497727	STM1417	*ssaP*	Secretion system apparatus	1.2	++	1.3				0.7
1497708..1498676	STM1418	*ssaQ*	Secretion system apparatus	1.2			0.8		0.8	0.3
1498744..1499391	STM1419	*ssaR*	Secretion system apparatus: homology with YscR of the secretion system of *Yersinia*	2.2	3.2					
1499388..1499654	STM1420	*ssaS*	Secretion system apparatus: homology with YscS of the secretion system of *Yersinia*		++					
1499655..1500434	STM1421	*ssaT*	Secretion system apparatus: homology with YscT of the secretion system of *Yersinia*		2.6			1.4		
1500431..1501489	STM1422	*ssaU*	Secretion system apparatus: homology with YscU of the secretion system of *Yersinia*		++					
1493656..1495701	STM1414	*ssaV*	Secretion system apparatus: homology with the LcrD family of proteins		2.2	2.5		1.4		
1484521..1484994	STM1399	*sscA*	Secretion system chaparone		++	1.5				
1487525..1487959	STM1403	*sscB*	Secretion system chaparone		++					
1476167..1476805	STM1391	*ssrB*	secretion system regulator, transcriptonal activator, homologous with degU/uvrY/bvgA, regulates SPI-2	2.2	3	5.1		3.7	4.7	0.7
208383..208922	STM0177	*stiA*	putative fimbrial subunit				1.6			

205079..207625	STM0175	*stiC*	putativie fimbrial usher	0.7	0.8	0.7	0.4	0.6	0.6	0.2	
2926802..2927854	STM2780	*STM2780*	homologue of pipB, putative pentapeptide repeats (8 copies)			2.7	1.2	1.4			0.6
104470..105756	STM0092	*surA*	peptidyl-prolyl cis-trans isomerase, survival protein, acting on folding of enveloppe proteins		0.6	2			0.8		
501665..502963	STM0447	*tig*	peptidyl-prolyl cis/trans isomerase, trigger factor; a molecular chaperone involved in cell division	0.6	0.6	0.4	0.4	0.4	0.4		0.8
1831260..1831988	STM1737	*tonB*	energy transducer; uptake of iron, cyanocobalimin; sensitivity to phages, colicins, putative binding site for fur		0.2	2.9			0.3		
4290265..4291032	STM4081	*tpiA*	triosephosphate isomerase		0.5	0.3	0.7	1.4			1.5
1895070..1896782	STM1796	*treA*	trehalase, periplasmic, stationary phase	1.6	1.9		2.5	0.5	1.6		
1470433..1471185	STM1385	*ttrB*	Tetrathionate reductase complex, subunit B		4.2						
1469410..1470432	STM1384	*ttrC*	Tetrathionate reductase complex, subunit C			0.6					
1473084..1473722	STM1387	*ttrR*	Tetrathionate reductase complex: response regulator	0.5							
1471349..1473127	STM1386	*ttrS*	Tetrathionate reductase complex: sensory transduction histidine kinase	0.6							
3990554..3991144	STM3790	*uhpA*	response regulator (repressor) in two-component system with UhpB, regulates uhpT operon (LuxR/UhpA family)			0.5		0.3	0.3	0.2	0.6
3989052..3990554	STM3789	*uhpB*	sensory histidine kinase in two-component regulatory sytem with UhpA	0.8							
259355..260113	STM0221	*upps*	undecaprenyl pyrophosphate synthetase, sigma E regulon transcribed as a three gene operon		0.6	0.3	0.6	0.6	0.4		
3760855..3761289	STM3591	*uspA*	universal stress protein A	1.9	1.6	7.2	3.1	2.3	2.1	4.1	1.6
3760132..3760467	STM3590	*uspB*	universal stress protein B, involved in stationary-phase resistance to ethanol	1.7	2.1	24.6	6.1	3.3	2.9	16.9	0.5
2038247..2038903	STM1947	*uvrY*	putative response regulator (LuxR/UhpA familiy)	0.8	0.8	0.4	0.8	1.6	2		1.3
4611064..4613502	STM4368	*vacB*	putative exoribonuclease		2	4.2	2.6	1.5	1.6		
2928373..2929302	STM2781	*virK*	virulence gene; homologous sequence to virK in Shigella	1.6	2.9	0.1		21.6	25.2		0.8
1205223..1205819	STM1119	*wraB (wrbA)*	trp-repressor binding protein, stationairy phase protein (trp = tryptophan pathway)	1.6	2.3	2.4		5.8	3.9	13.4	2
1380942..1381748	STM1302	*xthA*	exonuclease III, may repair singlet oxygen induced lesions (stationary phase oxygen stress resistance)	0.8	1.4	7.9	5.1	1.1	1.4		0.9
260995..262347	STM0223	*yaeL*	putative membrane-associated Zn-dependent protease, sigma E regulon, down-regulates rpoH and rpoE	0.7	0.7	0.3			0.6		0.1
415676..415951	STM0366	*yahO*	putative periplasmic protein	1.1	2.2	2.2	3.1	2.5	2.1	27.4	
677372..677800	STM0614	*ybdQ*	putative Universal stress protein UspA	1.6	1.9	7.6	3.4	1.8	2		1.6
891007..891273	STM0825	*ybiI*	hypothetical protein							17.3	
1825830..1826333	STM1729	*yciF*	putative cytoplasmic protein							6.5	
2258902..2260071	STM2164	*yehY*	putative ABC-type proline/glycine betain							8	
2501740..2502024	STM2390	*yfcZ*	putative cytoplasmic protein							0.2	
2807668..2808006	STM2665	*yfiA*	ribosome associated factor, stabilizes ribosomes against dissociation	1.5	2.1	29.1	64.1	18.7	13.1	51.2	2.3
2945151..2945600	STM2795	*ygaU*	putative LysM domain	1.1	2	4.2	5.9	6.6	3.3	++	0.8
3266716..3267435	STM3107	*yggN*	putative periplasmic protein	0.7		1.6		1.9	2.6		0.7
3485866..3486153	STM3321	*yhbH*	putative sigma N modulation factor	2	2	5.9	4.6	3.5	2.8	6.2	0.5
3489612..3490265	STM3327	*yhbL*	sigma cross-reacting protein 27A to sigma D and sigma H (SCRP-27A)	1.2	1.1	2.7	2.5	1.3	1.4		

3746509..3746754	STM3578	*yhhP*	small ubiquitous protein required for normal growth			0.5					
3779468..3780070	STM3606	*yhjB*	putative transcriptional regulator (LuxR/UhpA familiy)			2.6		2.1			
3780701..3781600	STM3607	*yhjC*	putative transcriptional regulator, LysR family				0.7	1.3	1		
3783052..3784374	STM3609	*yhjE*	putative MFS family transport protein	0.8		1.7		1.4			
3786584..3787351	STM3611	*yhjH*	putative diguanylate cyclase/phosphodiesterase domain 3, flagellar regulon related						1.5		
3828491..3829195	STM3641	*yhjY*	putative lipase		2.7						
3915683..3915916	STM3720	*yibR*	putative inner membrane protein	1.3				1.5			1.3
3927521..3928384	STM3735	*yicC*	putative stress-induced protein	0.8	0.7	1.5	1.9		0.9		
4614875..4615573	STM4371	*yjfJ*	putative Phage shock protein A, IM30, suppresses sigma 54-dependent transcription	1.7	3.2	2.4					
4729769..4730788	STM4486	*yjgB*	putative alcohol dehydrogenase		1.6	2		1.4	1.7		
1746131..1746565	STM1652	*ynaF*	putative universal stress protein	2	1.5	2.2	9.2	0.4	0.9		3.5
1676097..1676762	STM1588	*yncC*	putative regulatory protein, gntR family		2.6	1.4	1.3	1.8	2.3		
2068412..2068639	STM1984	*yodD*	putative cytoplasmic protein			3.5		2			
4642501..4643058	STM4405	*ytfJ*	putative transcriptional regulator	1.3				2.5			

Empty slot indicates no significant change in gene expression

++, Expression below detection limit in non-stressing conditions and highly up-regulated in stressing conditions
