# Supplementary material for: Non-essential genes form the hubs of genome scale protein function and environmental gene expression networks in Salmonella enterica serovar Typhimurium
Source: BMC Microbiol. 2013 Dec 17;13:294. doi: 10.1186/1471-2180-13-294 (PMC3878590; doi:10.1186/1471-2180-13-294)
Supplement: Additional file 2: Table S2 — Hubs or highly connected genes to culture conditions in the transcriptional network of S.Typhimurium, i.e. genes differentially transcribed under heat, oxidative, acid and/or osmotic stress and/or anaerobic condition, lag phase, exponential growth, stationary phase and immobilization. [file 1471-2180-13-294-S2.pdf]

Environmental Hubs
Hubs = Highly connected genes to culture conditions in the transcriptional network of *S.* Typhimurium, i.e. genes differentially transcribed under heat, oxidative, acid and/or osmotic stress and/or anaerobic condition, lag phase, exp growth, stat phase and immobilization
54 hubs: from 4 to 8 links

Locus Tag (LT2)	Gene name	degree (num links)	Condition and transcription pattern with respect to control conditions								
			Acid stress	Heat stress	Osmotic stress	Oxidative stress	Anaerobiosis	Immobilization	Exponential Growth	Stationary Phase	Lag Phase
STM2795	*ygaU*	**8**	Up	Up	Up	Up	Down	Up	Down	Up	
STM1563	*osmC*	**7**	Up	Up	Up	Up		Up	Down	Up	
STM3591	*uspA*	**7**	Up	Up	Up	Up	Up		Down	Up	
STM1119	*wraB*	**7**	Up	Up	Up	Up	Up		Down	Up	
STM1112	*cbpA*	**6**	Up	Down		Up	Up		Down	Up	
STM2559	*cadA*	**6**	Up	Down	Down	Up	Up		Down		
STM3385	*fis*	**6**	Down	Down	Down	Down	Down		Up		
STM3150	*hypO*	**6**	Down	Up	Down	Down	Up		Down		
STM4561	*osmY*	**6**	Up	Up	Up	Up	Down		Down		
STM0559	*rfbI*	**6**	Down	Down	Down	Down	Down			Down	
STM3590	*uspB*	**6**	Up	Up	Up	Up	Down			Up	
STM2665	*yfiA*	**6**	Up	Up	Up	Up	Up		Down		
STM1925	*flhD*	**5**	Down	Up		Down	Up	Down			
STM1318	*katE*	**5**	Up	Up	Up	Up		Up			
STM1705	*osmB*	**5**	Up		Up	Up	Down	Up			
STM2095	*rfbA*	**5**	Down	Down	Down	Down	Down				
STM2097	*rfbB*	**5**	Down	Down	Down	Down	Down				
STM2094	*rfbC*	**5**	Down	Down	Down	Down	Down				
STM2096	*rfbD*	**5**	Down	Down	Down	Down	Down				
STM2092	*rfbF*	**5**	Down	Down	Down	Down	Down				
STM2091	*rfbG*	**5**	Down	Down	Down	Down	Down				
STM2924	*rpoS*	**5**	Up	Up	Up	Up	Down				
STM0366	*yahO*	**5**	Up	Up	Up	Up				Up	
STM3321	*yhbH*	**5**	Up	Up	Up	Up	Down				
STM0066	*carA*	**4**	Down		Down	Down	Down				
STM2660	*clpB*	**4**		Up	Up	Up	Up				
STM1983	*dsrB*	**4**	Up		Up	Up	Down				
STM3453	*fkpA*	**4**	Up	Down		Down			Up		

STM0087	*folA*	4		Down	Down		Down	Down		
STM0487.S	*htpG*	4		Up	Up	Down	Up			
STM2925	*nlpD*	4	Up		Up	Up	Down			
STM2232	*oafA*	4	Down				Down	Down		Down
STM1928	*otsA*	4	Up	Up		Up		Up		
STM1929	*otsB*	4	Up		Up	Up		Up		
STM1088	*pipB*	4	Up	Up	Up	Up				
STM4291	*mrA (basR*	4	Up	Down		Down	Down			
STM2873	*prgI*	4	Down	Down			Up	Down		
STM4290	*proP*	4	Up	Up	Up	Up				
STM4175	*purD*	4	Down	Down		Down	Down			
STM1982	*rcsA*	4	Up			Up	Down	Up		
STM2090	*rfbH*	4	Down	Down	Down	Down				
STM2082	*rfbP*	4	Down	Down		Down	Down			
STM3415	*rpoA*	4	Down		Down	Down	Down			
STM4154	*rpoC*	4	Down	Down	Down	Down				
STM3568	*rpoH*	4	Up	Up		Up	Up			
STM2879	*sicP*	4		Up	Up		Down	Down		
STM2885	*sipB*	4	Down	Up			Up	Down		
STM2866	*sprB*	4	Up			Up	Up	Down		
STM2878	*sptP*	4	Down	Up			Down	Down		
STM0175	*stiC*	4	Down	Down	Down	Down				
STM0447	*tig*	4	Down	Down		Down	Down			
STM1947	*uvrY*	4	Up	Down		Down	Up			
STM0614	*ybdQ*	4	Up	Up		Up	Up			
STM1652	*ynaF*	4	Down	Up		Up	Up			
