# Supplementary material for: Non-essential genes form the hubs of genome scale protein function and environmental gene expression networks in Salmonella enterica serovar Typhimurium
Source: BMC Microbiol. 2013 Dec 17;13:294. doi: 10.1186/1471-2180-13-294 (PMC3878590; doi:10.1186/1471-2180-13-294)
Supplement: Additional file 3: Table S3 — Hubs or highly connected genes to cellular functions and metabolic pathways in the genome scale network for S. Typhimurium [file 1471-2180-13-294-S3.pdf]

Functional Hubs

Hubs= Highly connected genes to cellular functions and metabolic pathways in the genome scale network for S. Typhimurium

114 hubs: from 5 to 8 links

Locus Tag (LT2)	Gene name	degree (num links)	Cell function or metabolic pathway: first 3 letters for database
STM1769	*ychN*	8	COG_Inorganic_ion_transport_metabolism, TIGR_Cell_envelope_Uncategorized, TIGR_Cellular_processes_Cell_division, TIGR_DNA_metabolism_DNA_replication_recombination_repair, TIGR_Protein_fate_Degradation_proteins_peptides_glycopeptides, TIGR_Protein_fate_Protein_folding_stabilization, TIGR_Protein_synthesis_Ribosomal_proteins_synthesis_modification, TIGR_Regulatory_functions_Uncategorized
STM4262	*siiF*	8	COG_Defense_mechanisms, TIGR_Cell_envelope_Uncategorized, TIGR_Cellular_processes_Cell_division, TIGR_DNA_metabolism_DNA_replication_recombination_repair, TIGR_Protein_fate_Degradation_proteins_peptides_glycopeptides, TIGR_Protein_fate_Protein_folding_stabilization, TIGR_Protein_synthesis_Ribosomal_proteins_synthesis_modification, TIGR_Regulatory_functions_Uncategorized
STM0411	*yajD*	7	TIGR_Cell_envelope_Surface_structures, TIGR_Cellular_processes_Cell_division, TIGR_DNA_metabolism_DNA_replication_recombination_repair, TIGR_Protein_fate_Degradation_proteins_peptides_glycopeptides, TIGR_Protein_fate_Protein_folding_stabilization, TIGR_Protein_synthesis_Ribosomal_proteins_synthesis_modification, TIGR_Regulatory_functions_Uncategorized
STM0642	*ybeB*	7	TIGR_Cell_envelope_Surface_structures, TIGR_Cellular_processes_Cell_division, TIGR_DNA_metabolism_DNA_replication_recombination_repair, TIGR_Protein_fate_Degradation_proteins_peptides_glycopeptides, TIGR_Protein_fate_Protein_folding_stabilization, TIGR_Protein_synthesis_Ribosomal_proteins_synthesis_modification, TIGR_Regulatory_functions_Uncategorized
STM0766	*dcoC*	7	COG_Energy_production_conversion, KEGG_Amino_acid_Metabolism, KEGG_Carbohydrate_Metabolism, TIGR_Biosynthesis_cofactors_Heme_porphyrin_cobalamin, TIGR_Central_intermediary_metabolism_Uncategorized, TIGR_Energy_metabolism_Amino_acids_amines, TIGR_Purines_pyrimidines_nucleosides_nucleotides_Pyrimidine_ribonucleotide_biosynthesis
STM1211	*ndh*	7	COG_Energy_production_conversion, KEGG_Energy_Metabolism, TIGR_Central_intermediary_metabolism_Uncategorized, TIGR_Energy_metabolism_Electron_transport, TIGR_Energy_metabolism_Fermentation, TIGR_Energy_metabolism_Glycolysis, TIGR_Energy_metabolism_Uncategorized
STM1850	*yebU*	7	COG_Translation, TIGR_Amino_acid_biosynthesis_Serine_family, TIGR_Biosynthesis_cofactors_Heme_porphyrin_cobalamin, TIGR_Central_intermediary_metabolism_Uncategorized, TIGR_DNA_metabolism_DNA_replication_recombination_repair, TIGR_DNA_metabolism_Restriction_modification, TIGR_Protein_synthesis_tRNA_rRNA_base_modification
STM2322	*nuoH*	7	COG_Energy_production_conversion, KEGG_Energy_Metabolism, TIGR_Central_intermediary_metabolism_Uncategorized, TIGR_Energy_metabolism_Electron_transport, TIGR_Energy_metabolism_Fermentation, TIGR_Energy_metabolism_Glycolysis, TIGR_Energy_metabolism_Uncategorized
STM3220	*ygjO*	7	COG_Translation, TIGR_Amino_acid_biosynthesis_Serine_family, TIGR_Biosynthesis_cofactors_Heme_porphyrin_cobalamin, TIGR_Central_intermediary_metabolism_Uncategorized, TIGR_DNA_metabolism_DNA_replication_recombination_repair, TIGR_DNA_metabolism_Restriction_modification, TIGR_Protein_synthesis_tRNA_rRNA_base_modification
STM3353	*oadG*	7	COG_Energy_production_conversion, KEGG_Amino_acid_Metabolism, KEGG_Carbohydrate_Metabolism, TIGR_Biosynthesis_cofactors_Heme_porphyrin_cobalamin, TIGR_Central_intermediary_metabolism_Uncategorized, TIGR_Energy_metabolism_Amino_acids_amines, TIGR_Purines_pyrimidines_nucleosides_nucleotides_Salvage_nucleosides_nucleotides

STM4129	*trmA*	7	COG_Translation, TIGR_Amino_acid_biosynthesis_Uncategorized, TIGR_Biosynthesis_cofactors_Heme_porphyrin_cobalamin, TIGR_Central_intermediary_metabolism_Uncategorized, TIGR_DNA_metabolism_DNA_replication_recombination_repair, TIGR_DNA_metabolism_Restriction_modification, TIGR_Protein_synthesis_tRNA_rRNA_base_modification
STM4500	*yjhP*	7	COG_Coenzyme_transport_metabolism, TIGR_Amino_acid_biosynthesis_Uncategorized, TIGR_Biosynthesis_cofactors_Heme_porphyrin_cobalamin, TIGR_Central_intermediary_metabolism_Uncategorized, TIGR_DNA_metabolism_DNA_replication_recombination_repair, TIGR_DNA_metabolism_Restriction_modification, TIGR_Protein_synthesis_Uncategorized
STM0545	*fimC*	6	COG_Cell_motility, COG_Intracellular_trafficking_secretion, KEGG_Cell_motility_Pilus, KEGG_Chaperones, TIGR_Cell_envelope_Uncategorized, TIGR_Protein_fate_Protein_folding_stabilization
STM0577	*STM0577*	6	COG_Carbohydrate_transport_metabolism, KEGG_Carbohydrate_Metabolism, KEGG_Phosphotransferase_System, TIGR_Transport_binding_proteins_Amino_acids_peptides_amines, TIGR_Transport_binding_proteins_Cations_iron_carrying_compounds, TIGR_Transport_binding_proteins_Uncategorized
STM0857	*acd-6*	6	COG_Lipid_transport_metabolism, KEGG_Amino_acid_Metabolism, KEGG_Carbohydrate_Metabolism, KEGG_Lipid_Metabolism, KEGG_Other_Amino_acids_Metabolism, TIGR_Fatty_acid_phospholipid_metabolism_Degradation
STM1080	*yccW*	6	TIGR_Amino_acid_biosynthesis_Serine_family, TIGR_Biosynthesis_cofactors_Heme_porphyrin_cobalamin, TIGR_Central_intermediary_metabolism_Uncategorized, TIGR_DNA_metabolism_DNA_replication_recombination_repair, TIGR_DNA_metabolism_Restriction_modification, TIGR_Protein_synthesis_tRNA_rRNA_base_modification
STM1209	*nagZ*	6	COG_Carbohydrate_transport_metabolism, KEGG_Carbohydrate_Metabolism, TIGR_Cellular_processes_Toxin_production_resistance, TIGR_Central_intermediary_metabolism_Uncategorized, TIGR_Energy_metabolism_Biosynthesis_degradation_polysaccharides, TIGR_Fatty_acid_phospholipid_metabolism_Degradation
STM1391	*ssrB*	6	COG_Transcription, COG_Signal_transduction_mechanisms, TIGR_Cellular_processes_Toxin_production_resistance, TIGR_Regulatory_functions_DNA_interactions, TIGR_Regulatory_functions_Uncategorized, TIGR_Signal_transduction_Two_component_systems
STM1525	*yneH*	6	COG_Amino_acid_transport_metabolism, KEGG_Amino_acid_Metabolism, KEGG_Other_Amino_acids_Metabolism, TIGR_Energy_metabolism_Amino_acids_amines, TIGR_Energy_metabolism_Uncategorized, TIGR_Purines_pyrimidines_nucleosides_nucleotides_Pyrimidine_ribonucleotide_biosynthesis
STM1532	*STM1532*	6	COG_Energy_production_conversion, TIGR_Central_intermediary_metabolism_Uncategorized, TIGR_Energy_metabolism_Electron_transport, TIGR_Energy_metabolism_Fermentation, TIGR_Energy_metabolism_Glycolysis, TIGR_Energy_metabolism_Uncategorized
STM1616	*STM1616*	6	COG_Signal_transduction_mechanisms, COG_Carbohydrate_transport_metabolism, KEGG_Carbohydrate_Metabolism, KEGG_Phosphotransferase_System, TIGR_Signal_transduction_PTS, TIGR_Transport_binding_proteins_Carbohydrates_organic_alcohols_acids
STM1876	*holE*	6	KEGG_DNA_repair, KEGG_DNA_replication, KEGG_Nucleotide_Metabolism, TIGR_DNA_metabolism_DNA_replication_recombination_repair, TIGR_Transcription_DNA_dependent_RNA_polymerase, TIGR_Transcription_RNA_processing
STM4385	*ptxA*	6	COG_Signal_transduction_mechanisms, COG_Carbohydrate_transport_metabolism, KEGG_Carbohydrate_Metabolism, KEGG_Phosphotransferase_System, TIGR_Signal_transduction_PTS, TIGR_Transport_binding_proteins_Carbohydrates_organic_alcohols_acids
STM2388	*fadJ*	6	COG_Lipid_transport_metabolism, KEGG_Amino_acid_Metabolism, KEGG_Carbohydrate_Metabolism, KEGG_Lipid_Metabolism, KEGG_Other_Amino_acids_Metabolism, TIGR_Fatty_acid_phospholipid_metabolism_Degradation
STM2555	*glyA*	6	COG_Amino_acid_transport_metabolism, KEGG_Amino_acid_Metabolism, KEGG_Cofactors_Vitamins_Metabolism, TIGR_Amino_acid_biosynthesis_Serine_family, TIGR_Biosynthesis_cofactors_Folic_acid, TIGR_Purines_pyrimidines_nucleosides_nucleotides_Pyrimidine_ribonucleotide_biosynthesis

STM2935	*cysD*	6	COG_Amino_acid_transport_metabolism, COG_Coenzyme_transport_metabolism, KEGG_Energy_Metabolism, KEGG_Nucleotide_Metabolism, KEGG_Other_Amino_acids_Metabolism, TIGR_Central_intermediary_metabolism_Uncategorized
STM3056	*visC*	6	COG_Energy_production_conversion, COG_Coenzyme_transport_metabolism, TIGR_Cellular_processes_Pathogenesis, TIGR_Central_intermediary_metabolism_Nitrogen_metabolism, TIGR_Energy_metabolism_Electron_transport, TIGR_Energy_metabolism_Sugars
STM3080	*STM3080*	6	COG_Carbohydrate_transport_metabolism, TIGR_Central_intermediary_metabolism_Uncategorized, TIGR_Energy_metabolism_Electron_transport, TIGR_Energy_metabolism_Fermentation, TIGR_Energy_metabolism_Glycolysis, TIGR_Energy_metabolism_Uncategorized
STM3250	*garD*	6	COG_Carbohydrate_transport_metabolism, TIGR_Central_intermediary_metabolism_Uncategorized, TIGR_Energy_metabolism_Electron_transport, TIGR_Energy_metabolism_Fermentation, TIGR_Energy_metabolism_Glycolysis, TIGR_Energy_metabolism_Uncategorized
STM3257	*STM3257*	6	COG_Carbohydrate_transport_metabolism, KEGG_Carbohydrate_Metabolism, TIGR_Central_intermediary_metabolism_Amino_sugars, TIGR_Energy_metabolism_Glycolysis, TIGR_Energy_metabolism_TCA_cycle, TIGR_Energy_metabolism_Uncategorized
STM3258	*STM3258*	6	COG_Signal_transduction_mechanisms, COG_Carbohydrate_transport_metabolism, KEGG_Carbohydrate_Metabolism, KEGG_Phosphotransferase_System, TIGR_Signal_transduction_PTS, TIGR_Transport_binding_proteins_Carbohydrates_organic_alcohols_acids
STM3385	*fis*	6	COG_Transcription, COG_Replication_recombination_repair, KEGG_DNA_repair, KEGG_DNA_replication, KEGG_Transcription, TIGR_DNA_metabolism_DNA_replication_recombination_repair
STM3593	*yhiQ*	6	TIGR_Amino_acid_biosynthesis_Serine_family, TIGR_Biosynthesis_cofactors_Heme_porphyrin_cobalamin, TIGR_Central_intermediary_metabolism_Uncategorized, TIGR_DNA_metabolism_DNA_replication_recombination_repair, TIGR_DNA_metabolism_Restriction_modification, TIGR_Protein_synthesis_tRNA_rRNA_base_modification
STM3742	*spoT*	6	COG_Transcription, COG_Signal_transduction_mechanisms, KEGG_Nucleotide_Metabolism, TIGR_Cellular_processes_Adaptations_to_atypical_conditions, TIGR_Purines_pyrimidines_nucleosides_nucleotides_Purine_ribonucleotide_biosynthesis, TIGR_Regulatory_functions_Uncategorized
STM3983	*fadB*	6	COG_Lipid_transport_metabolism, KEGG_Amino_acid_Metabolism, KEGG_Carbohydrate_Metabolism, KEGG_Lipid_Metabolism, KEGG_Other_Amino_acids_Metabolism, TIGR_Fatty_acid_phospholipid_metabolism_Degradation
STM4034	*fdhE*	6	COG_Posttranslational_modification_protein_turnover_chaperones, TIGR_Central_intermediary_metabolism_Uncategorized, TIGR_Energy_metabolism_Electron_transport, TIGR_Energy_metabolism_Fermentation, TIGR_Energy_metabolism_Glycolysis, TIGR_Energy_metabolism_Uncategorized
STM4343	*frdA*	6	COG_Energy_production_conversion, KEGG_Carbohydrate_Metabolism, KEGG_Energy_Metabolism, TIGR_Energy_metabolism_Anaerobic, TIGR_Energy_metabolism_Electron_transport, TIGR_Energy_metabolism_Uncategorized
PSLT068	*PSLT068*	5	KEGG_Cell_motility, KEGG_DNA_replication, KEGG_Transcription, TIGR_DNA_metabolism_DNA_replication_recombination_repair, TIGR_Transcription_Degradation_RNA
STM3352	*oadA*	5	COG_Energy_production_conversion, KEGG_Amino_acid_Metabolism, KEGG_Carbohydrate_Metabolism, TIGR_Energy_metabolism_Pentose_phosphate_pathway, TIGR_Transport_binding_proteins_Cations_iron_carrying_compounds
STM0066	*carA*	5	COG_Amino_acid_transport_metabolism, COG_Nucleotide_transport_metabolism, KEGG_Amino_acid_Metabolism, KEGG_Nucleotide_Metabolism, TIGR_Purines_pyrimidines_nucleosides_nucleotides_Pyrimidine_ribonucleotide_biosynthesis
STM0067	*carB*	5	COG_Amino_acid_transport_metabolism, COG_Nucleotide_transport_metabolism, KEGG_Amino_acid_Metabolism, KEGG_Nucleotide_Metabolism, TIGR_Purines_pyrimidines_nucleosides_nucleotides_Pyrimidine_ribonucleotide_biosynthesis
STM0116	*ilvI*	5	COG_Amino_acid_transport_metabolism, COG_Coenzyme_transport_metabolism, KEGG_Amino_acid_Metabolism, KEGG_Cofactors_Vitamins_Metabolism, TIGR_Amino_acid_biosynthesis_Pyruvate_family
STM0231	*dnaE*	5	COG_Replication_recombination_repair, KEGG_DNA_repair, KEGG_DNA_replication, KEGG_Nucleotide_Metabolism, TIGR_DNA_metabolism_DNA_replication_recombination_repair

STM0264	*dnaQ*	5	COG_Replication_recombination_repair, KEGG_DNA_repair, KEGG_DNA_replication, KEGG_Nucleotide_Metabolism, TIGR_DNA_metabolism_DNA_replication_recombination_repair
STM0340	*stbA*	5	COG_Cell_motility, COG_Intracellular_trafficking_secretion, KEGG_Cell_motility_Pilus, TIGR_Cell_envelope_Surface_structures, TIGR_Cell_envelope_Uncategorized
STM2430	*cysK*	5	COG_Amino_acid_transport_metabolism, KEGG_Amino_acid_Metabolism, KEGG_Energy_Metabolism, KEGG_Other_Amino_acids_Metabolism, TIGR_Amino_acid_biosynthesis_Serine_family
STM0484	*dnaX*	5	COG_Replication_recombination_repair, KEGG_DNA_repair, KEGG_DNA_replication, KEGG_Nucleotide_Metabolism, TIGR_DNA_metabolism_DNA_replication_recombination_repair
STM0512	*sfbC*	5	COG_Inorganic_ion_transport_metabolism, KEGG_ABC_transporters_Phosphate_amino_acid, TIGR_Transport_binding_proteins_Amino_acids_peptides_amines, TIGR_Transport_binding_proteins_Cations_iron_carrying_compounds, TIGR_Transport_binding_proteins_Uncategorized
STM0595	*entC*	5	COG_Coenzyme_transport_metabolism, COG_Secondary_metabolites_biosynthesis_transport_catabolism, KEGG_Cofactors_Vitamins_Metabolism, TIGR_Biosynthesis_cofactors_Menaquinone_ubiquinone, TIGR_Biosynthesis_cofactors_Pantothenate_coenzyme_A
STM0646	*holA*	5	COG_Replication_recombination_repair, KEGG_DNA_repair, KEGG_DNA_replication, KEGG_Nucleotide_Metabolism, TIGR_DNA_metabolism_DNA_replication_recombination_repair
STM0861	*yliI*	5	TIGR_Central_intermediary_metabolism_Uncategorized, TIGR_Energy_metabolism_Electron_transport, TIGR_Energy_metabolism_Fermentation, TIGR_Energy_metabolism_Glycolysis, TIGR_Energy_metabolism_Uncategorized
STM0968	*ycaD*	5	COG_Carbohydrate_transport_metabolism, KEGG_Major_facilitator_superfamily_Protein_transporters, TIGR_Transport_binding_proteins_Amino_acids_peptides_amines, TIGR_Transport_binding_proteins_Cations_iron_carrying_compounds, TIGR_Transport_binding_proteins_Uncategorized
STM0977	*serC*	5	COG_Amino_acid_transport_metabolism, COG_Coenzyme_transport_metabolism, KEGG_Amino_acid_Metabolism, KEGG_Cofactors_Vitamins_Metabolism, TIGR_Amino_acid_biosynthesis_Serine_family
STM1016	*STM1016*	5	TIGR_Regulatory_functions_Uncategorized
STM1135	*ycdW*	5	COG_Amino_acid_transport_metabolism, COG_Coenzyme_transport_metabolism, KEGG_Amino_acid_Metabolism, KEGG_Carbohydrate_Metabolism, TIGR_Central_intermediary_metabolism_Uncategorized
STM1171	*flgN*	5	COG_Cell_motility, COG_Intracellular_trafficking_secretion, COG_Posttranslational_modification_protein_turnover_chaperones, KEGG_Cell_motility_Flagellar, TIGR_Cell_envelope_Uncategorized
STM1172	*flgM*	5	COG_Transcription, COG_Cell_motility, COG_Intracellular_trafficking_secretion, KEGG_Cell_motility_Flagellar, TIGR_Cell_envelope_Uncategorized
STM1182	*flgJ*	5	COG_Cell_wall_membrane_biogenesis, COG_Cell_motility, COG_Posttranslational_modification_protein_turnover_chaperones, KEGG_Cell_motility_Flagellar, TIGR_Cell_envelope_Biosynthesis_degradation_murein_sacculus_peptidoglycan
STM1201	*holB*	5	COG_Replication_recombination_repair, KEGG_DNA_repair, KEGG_DNA_replication, KEGG_Nucleotide_Metabolism, TIGR_DNA_metabolism_DNA_replication_recombination_repair
STM1203	*ptsG*	5	COG_Carbohydrate_transport_metabolism, KEGG_Carbohydrate_Metabolism, KEGG_Phosphotransferase_System, TIGR_DNA_metabolism_Restriction_modification, TIGR_Protein_fate_Degradation_proteins_peptides_glycopeptides
STM1392	*ssrA*	5	COG_Signal_transduction_mechanisms, KEGG_Carbohydrate_Metabolism, KEGG_Nucleotide_Metabolism, TIGR_Energy_metabolism_Anaerobic, TIGR_Regulatory_functions_Uncategorized
STM1444	*slyA*	5	COG_Transcription, KEGG_Transcription, TIGR_Cellular_processes_Toxin_production_resistance, TIGR_Protein_fate_Degradation_proteins_peptides_glycopeptides, TIGR_Protein_fate_Protein_peptide_secretion_trafficking
STM1486	*ynfM*	5	COG_Carbohydrate_transport_metabolism, KEGG_Major_facilitator_superfamily_Drug_transporters, TIGR_Transport_binding_proteins_Amino_acids_peptides_amines, TIGR_Transport_binding_proteins_Cations_iron_carrying_compounds, TIGR_Transport_binding_proteins_Uncategorized

STM1627	*adhC*	5	COG_Energy_production_conversion, KEGG_Amino_acid_Metabolism, KEGG_Carbohydrate_Metabolism, KEGG_Lipid_Metabolism, TIGR_Energy_metabolism_Fermentation
STM1749	*adhE*	5	COG_Energy_production_conversion, KEGG_Amino_acid_Metabolism, KEGG_Carbohydrate_Metabolism, KEGG_Lipid_Metabolism, TIGR_Energy_metabolism_Fermentation
STM1780	*prsA*	5	COG_Amino_acid_transport_metabolism, COG_Nucleotide_transport_metabolism, KEGG_Carbohydrate_Metabolism, KEGG_Nucleotide_Metabolism, TIGR_Purines_pyrimidines_nucleosides_nucleotides_Purine_ribonucleotide_biosynthesis
STM1917	*cheB*	5	COG_Signal_transduction_mechanisms, COG_Cell_motility, KEGG_Cell_motility_Flagellar, KEGG_Signal_Transduction, TIGR_Cellular_processes_Chemotaxis_motility
STM1954	*fliY*	5	COG_Signal_transduction_mechanisms, COG_Amino_acid_transport_metabolism, KEGG_ABC_transporters_Phosphate_amino_acid, KEGG_Cell_motility_Flagellar, TIGR_Transport_binding_proteins_Amino_acids_peptides_amines
STM1961	*fliS*	5	COG_Cell_motility, COG_Intracellular_trafficking_secretion, COG_Posttranslational_modification_protein_turnover_chaperones, KEGG_Cell_motility_Flagellar, TIGR_Cellular_processes_Chemotaxis_motility
STM1973	*fliJ*	5	COG_Cell_motility, COG_Intracellular_trafficking_secretion, COG_Posttranslational_modification_protein_turnover_chaperones, KEGG_Cell_motility_Flagellar, TIGR_Cellular_processes_Chemotaxis_motility
STM2314	*STM2314*	5	COG_Signal_transduction_mechanisms, COG_Cell_motility, KEGG_Cell_motility_Flagellar, KEGG_Signal_Transduction, TIGR_Cellular_processes_Chemotaxis_motility
STM2338	*pta*	5	COG_Energy_production_conversion, KEGG_Carbohydrate_Metabolism, TIGR_Central_intermediary_metabolism_Uncategorized, TIGR_Energy_metabolism_Fermentation, TIGR_Energy_metabolism_TCA_cycle
STM2343	*STM2343*	5	COG_Carbohydrate_transport_metabolism, KEGG_Carbohydrate_Metabolism, KEGG_Phosphotransferase_System, TIGR_Signal_transduction_PTS, TIGR_Transport_binding_proteins_Carbohydrates_organic_alcohols_acids
STM2404	*STM2404*	5	COG_Inorganic_ion_transport_metabolism, TIGR_Regulatory_functions_Uncategorized, TIGR_Transport_binding_proteins_Anions, TIGR_Transport_binding_proteins_Cations_iron_carrying_compounds, TIGR_Transport_binding_proteins_Uncategorized
STM2430	*cysK*	5	COG_Amino_acid_transport_metabolism, KEGG_Amino_acid_Metabolism, KEGG_Energy_Metabolism, KEGG_Other_Amino_acids_Metabolism, TIGR_Amino_acid_biosynthesis_Serine_family
STM2440	*cysM*	5	COG_Amino_acid_transport_metabolism, KEGG_Amino_acid_Metabolism, KEGG_Energy_Metabolism, KEGG_Other_Amino_acids_Metabolism, TIGR_Amino_acid_biosynthesis_Serine_family
STM2624	*STM2624*	5	COG_Transcription, COG_Carbohydrate_transport_metabolism, TIGR_Mobile_extrachromosomal_element_functions_Prophage_functions, TIGR_Mobile_extrachromosomal_element_functions_Transposon_functions, TIGR_Regulatory_functions_Uncategorized
STM2758	*glcA*	5	COG_Carbohydrate_transport_metabolism, KEGG_Carbohydrate_Metabolism, KEGG_Phosphotransferase_System, TIGR_Signal_transduction_PTS, TIGR_Transport_binding_proteins_Carbohydrates_organic_alcohols_acids
STM2771	*fljB*	5	COG_Cell_motility, KEGG_Cell_motility_Flagellar, TIGR_Cell_envelope_Uncategorized, TIGR_Mobile_extrachromosomal_element_functions_Prophage_functions, TIGR_Transcription_RNA_processing
STM2792	*gabT*	5	COG_Amino_acid_transport_metabolism, KEGG_Amino_acid_Metabolism, KEGG_Carbohydrate_Metabolism, KEGG_Other_Amino_acids_Metabolism, TIGR_Central_intermediary_metabolism_Uncategorized
STM2887	*spaS*	5	COG_Cell_motility, COG_Intracellular_trafficking_secretion, KEGG_Protein_Secretion, TIGR_Cell_envelope_Uncategorized, TIGR_Cellular_processes_Adaptations_to_atypical_conditions
STM2891	*spaO*	5	COG_Cell_motility, COG_Intracellular_trafficking_secretion, KEGG_Protein_Secretion, TIGR_Cell_envelope_Uncategorized, TIGR_Cellular_processes_Adaptations_to_atypical_conditions
STM2933	*cysC*	5	COG_Inorganic_ion_transport_metabolism, KEGG_Energy_Metabolism, KEGG_Nucleotide_Metabolism, KEGG_Other_Amino_acids_Metabolism, TIGR_Central_intermediary_metabolism_Uncategorized
STM2934	*cysN*	5	COG_Inorganic_ion_transport_metabolism, KEGG_Energy_Metabolism, KEGG_Nucleotide_Metabolism, KEGG_Other_Amino_acids_Metabolism, TIGR_Central_intermediary_metabolism_Uncategorized

STM3019	*yqeF*	5	COG_Lipid_transport_metabolism, KEGG_Amino_acid_Metabolism, KEGG_Carbohydrate_Metabolism, KEGG_Lipid_Metabolism, TIGR_Fatty_acid_phospholipid_metabolism_Uncategorized
STM1614	*gatC*	5	COG_Carbohydrate_transport_metabolism, KEGG_Carbohydrate_Metabolism, KEGG_Phosphotransferase_System, TIGR_Signal_transduction_PTS, TIGR_Transport_binding_proteins_Carbohydrates_organic_alcohols_acids
STM3339	*nanA*	5	COG_Cell_wall_membrane_biogenesis, COG_Amino_acid_transport_metabolism, KEGG_Carbohydrate_Metabolism, TIGR_Cell_envelope_Biosynthesis_degradation_surface_polysaccharides_lipopolysaccharides, TIGR_Central_intermediary_metabolism_Amino_sugars
STM3352	*oadA*	5	COG_Energy_production_conversion, KEGG_Amino_acid_Metabolism, KEGG_Carbohydrate_Metabolism, TIGR_Energy_metabolism_Pentose_phosphate_pathway, TIGR_Transport_binding_proteins_Cations_iron_carrying_compounds
STM3415	*rpoA*	5	COG_Transcription, KEGG_DNA_repair, KEGG_Nucleotide_Metabolism, KEGG_Transcription, TIGR_Transcription_DNA_dependent_RNA_polymerase
STM3473	*yhfC*	5	COG_Carbohydrate_transport_metabolism, KEGG_Major_facilitator_superfamily_Protein_transporters, TIGR_Transport_binding_proteins_Amino_acids_peptides_amines, TIGR_Transport_binding_proteins_Cations_iron_carrying_compounds, TIGR_Transport_binding_proteins_Uncategorized
STM3904	*ilvD*	5	COG_Carbohydrate_transport_metabolism, COG_Amino_acid_transport_metabolism, KEGG_Amino_acid_Metabolism, KEGG_Cofactors_Vitamins_Metabolism, TIGR_Amino_acid_biosynthesis_Pyruvate_family
STM3644	*bisC*	5	COG_Energy_production_conversion, TIGR_Biosynthesis_cofactors_Biotin, TIGR_Energy_metabolism_Anaerobic, TIGR_Protein_fate_Protein_modification_repair, TIGR_Regulatory_functions_Uncategorized
STM3741	*rpoZ*	5	COG_Transcription, KEGG_DNA_repair, KEGG_Nucleotide_Metabolism, KEGG_Transcription, TIGR_Transcription_DNA_dependent_RNA_polymerase
STM3772	*STM3772*	5	COG_Carbohydrate_transport_metabolism, KEGG_Carbohydrate_Metabolism, KEGG_Phosphotransferase_System, TIGR_Cellular_processes_Uncategorized, TIGR_Transport_binding_proteins_Carbohydrates_organic_alcohols_acids
STM3784	*STM3784*	5	COG_Signal_transduction_mechanisms, COG_Carbohydrate_transport_metabolism, KEGG_Carbohydrate_Metabolism, KEGG_Phosphotransferase_System, TIGR_Transport_binding_proteins_Carbohydrates_organic_alcohols_acids
STM3796	*ilvB*	5	COG_Amino_acid_transport_metabolism, COG_Coenzyme_transport_metabolism, KEGG_Amino_acid_Metabolism, KEGG_Cofactors_Vitamins_Metabolism, TIGR_Amino_acid_biosynthesis_Pyruvate_family
STM3837	*dnaN*	5	COG_Replication_recombination_repair, KEGG_DNA_repair, KEGG_DNA_replication, KEGG_Nucleotide_Metabolism, TIGR_DNA_metabolism_DNA_replication_recombination_repair
STM3901	*ilvG*	5	COG_Amino_acid_transport_metabolism, COG_Coenzyme_transport_metabolism, KEGG_Amino_acid_Metabolism, KEGG_Cofactors_Vitamins_Metabolism, TIGR_Amino_acid_biosynthesis_Pyruvate_family
STM3903	*ilvE*	5	COG_Amino_acid_transport_metabolism, COG_Coenzyme_transport_metabolism, KEGG_Amino_acid_Metabolism, KEGG_Cofactors_Vitamins_Metabolism, TIGR_Amino_acid_biosynthesis_Serine_family
STM3904	*ilvD*	5	COG_Carbohydrate_transport_metabolism, COG_Amino_acid_transport_metabolism, KEGG_Amino_acid_Metabolism, KEGG_Cofactors_Vitamins_Metabolism, TIGR_Amino_acid_biosynthesis_Pyruvate_family
STM3909	*ilvC*	5	COG_Amino_acid_transport_metabolism, COG_Coenzyme_transport_metabolism, KEGG_Amino_acid_Metabolism, KEGG_Cofactors_Vitamins_Metabolism, TIGR_Amino_acid_biosynthesis_Serine_family
STM3963	*yigM*	5	COG_Carbohydrate_transport_metabolism, COG_Amino_acid_transport_metabolism, TIGR_Transport_binding_proteins_Amino_acids_peptides_amines, TIGR_Transport_binding_proteins_Cations_iron_carrying_compounds, TIGR_Transport_binding_proteins_Uncategorized
STM3974	*tatB*	5	COG_Intracellular_trafficking_secretion, KEGG_Protein_Secretion, TIGR_Amino_acid_biosynthesis_Aromatic_family, TIGR_Energy_metabolism_TCA_cycle, TIGR_Transport_binding_proteins_Carbohydrates_organic_alcohols_acids
STM4044	*STM4044*	5	COG_Energy_production_conversion, KEGG_Amino_acid_Metabolism, KEGG_Carbohydrate_Metabolism, KEGG_Lipid_Metabolism, TIGR_Energy_metabolism_Fermentation

STM4153	*rpoB*	5	COG_Transcription, KEGG_DNA_repair, KEGG_Nucleotide_Metabolism, KEGG_Transcription, TIGR_Transcription_DNA_dependent_RNA_polymerase
STM4154	*rpoC*	5	COG_Transcription, KEGG_DNA_repair, KEGG_Nucleotide_Metabolism, KEGG_Transcription, TIGR_Transcription_DNA_dependent_RNA_polymerase
STM4229	*malE*	5	COG_Carbohydrate_transport_metabolism, KEGG_ABC_transporters_Oligosaccharide_polyol, TIGR_Biosynthesis_cofactors_Heme_porphyrin_cobalamin, TIGR_Protein_fate_Degradation_proteins_peptides_glycopeptides, TIGR_Regulatory_functions_Uncategorized
STM4237	*lexA*	5	COG_Transcription, COG_Signal_transduction_mechanisms, KEGG_DNA_repair, TIGR_DNA_metabolism_DNA_replication_recombination_repair, TIGR_Regulatory_functions_DNA_interactions
STM4259	*STM4259*	5	COG_Cell_wall_membrane_biogenesis, COG_Intracellular_trafficking_secretion, TIGR_Amino_acid_biosynthesis_Aromatic_family, TIGR_Energy_metabolism_TCA_cycle, TIGR_Transport_binding_proteins_Carbohydrates_organic_alcohols_acids
STM4285	*fdhF*	5	KEGG_Carbohydrate_Metabolism, TIGR_Energy_metabolism_Fermentation, TIGR_Energy_metabolism_Glycolysis, TIGR_Energy_metabolism_TCA_cycle, TIGR_Energy_metabolism_Uncategorized
STM4385	*ptxA*	5	COG_Signal_transduction_mechanisms, COG_Carbohydrate_transport_metabolism, KEGG_Carbohydrate_Metabolism, KEGG_Phosphotransferase_System, TIGR_Signal_transduction_PTS, TIGR_Transport_binding_proteins_Carbohydrates_organic_alcohols_acids
STM4476.S	*holC*	5	COG_Replication_recombination_repair, KEGG_DNA_repair, KEGG_DNA_replication, KEGG_Nucleotide_Metabolism, TIGR_DNA_metabolism_DNA_replication_recombination_repair
STM4557	*holD*	5	COG_Replication_recombination_repair, KEGG_DNA_repair, KEGG_DNA_replication, KEGG_Nucleotide_Metabolism, TIGR_DNA_metabolism_DNA_replication_recombination_repair
STM4564	*yjjV*	5	COG_Replication_recombination_repair, TIGR_Cellular_processes_Uncategorized, TIGR_Central_intermediary_metabolism_Uncategorized, TIGR_Energy_metabolism_Biosynthesis_degradation_polysaccharides, TIGR_Fatty_acid_phospholipid_metabolism_Degradation
