# Supplementary material for: Non-essential genes form the hubs of genome scale protein function and environmental gene expression networks in Salmonella enterica serovar Typhimurium
Source: BMC Microbiol. 2013 Dec 17;13:294. doi: 10.1186/1471-2180-13-294 (PMC3878590; doi:10.1186/1471-2180-13-294)
Supplement: Additional file 4: Table S4 — Plasmids and Phages used in DNA manipulations. [file 1471-2180-13-294-S4.pdf]

Plasmids and Phages used in DNA manipulations

Description	Name	Source
Plasmids		
	pKD46	Datsenko [72]
	pKD3	Datsenko [72]
	pCP20	Datsenko [72]
Phage		
	P22 (HT105/1 *int-201*)	Maloy [73]
