# Supplementary material for: Non-essential genes form the hubs of genome scale protein function and environmental gene expression networks in Salmonella enterica serovar Typhimurium
Source: BMC Microbiol. 2013 Dec 17;13:294. doi: 10.1186/1471-2180-13-294 (PMC3878590; doi:10.1186/1471-2180-13-294)
Supplement: Additional file 5: Table S5 — Sequnce of primers used in the study. [file 1471-2180-13-294-S5.pdf]

Table S5. Sequnce of primers used in the study

Primer	Sequence (5'⟶ 3')	Application in study
2,47	CGAATCGCTACACACCTGATTAATCTGAGGAGTAGTACAAGTGTAGGCTGGAGCTGCTTC	Fw – wraB deletion
2,48	TTTGCTTCTTGAGTTGGCATTCGAATCCTCCTGTTGAAGACATATGAATATCCTCCTTAG	Rv – wraB deletion
2,49	ACACTTTTCTTTGTGTGTATTTTTGACAAGCGAGGTGAATGTGTAGGCTGGAGCTGCTTC	Fw – ygaU deletion
2,5	GATGGCAGGGAAAATGTAGCCTGGCGCGACCTGATAGCCACATATGAATATCCTCCTTAG	Rv – ygaU deletion
2,51	AGCTCAATGGTCATCGACAACTTATGGAAGGAGTAACACTGTGTAGGCTGGAGCTGCTTC	Fw– uspA deletion
2,52	GACCAGACGCGGTCTTAGCCGCCAGCCGGCACGGCAAGTACATATGAATATCCTCCTTAG	Rv– uspA deletion
2,53	GGGGTCCCTTCTGTATGTATTGATTTAGCGAGATGATGCTGTGTAGGCTGGAGCTGCTTC	Fw– cbpA deletion
2,54	AACAAAATTCGGTGATGGTAAAGGTGACAGTGATGTTAGCCATATGAATATCCTCCTTAG	Rv– cbpA deletion
2,55	TGGTCTATACTTTATTTTTTGAGCCAACAGGAGAGCAAAAGTGTAGGCTGGAGCTGCTTC	Fw – osmC deletion
2,56	TCAGCCCGATCAACCCATCGTTACCGGGCTGGAGAGGTTACATATGAATATCCTCCTTAG	Rv– osmC deletion
2,57	CGCCATTGCCGACAAAATTTCTCAAGGAGAAGGGTTATCCGTGTAGGCTGGAGCTGCTTC	Fw – ychN deletion
2,58	GAAGTCTATCCTGACGCGGGAGCTTTTCCCGCGTACAGATCATATGAATATCCTCCTTAG	Rv – ychN deletion
2,59	ATAACGGAGGCCCCTCACCTTTGGGTGAGGGGGTTTACTTGTGTAGGCTGGAGCTGCTTC	Fw - STM4262 deletion
2,6	AAGCAGTACCACCTGATAACAGCGACAAGCGCTGCTTATTCATATGAATATCCTCCTTAG	Rv - STM4262 deletion
2,61	CTGGGCATAACCCTTTGTATTATTCAGGTACCTGTTAATTGTGTAGGCTGGAGCTGCTTC	Fw– yajD deletion
2,62	CTAACATTCAACAGCACCAACAGCTTTGTCAGCAGTCTGCCATATGAATATCCTCCTTAG	Rv– yajD deletion
2,63	TTCTATCGTTATAACGCAATTATTCACCCAGGGGGAAAACGTGTAGGCTGGAGCTGCTTC	Fw – ybeB deletion
2,64	AGTCGGGCATCTTCGTGCCGACAGCGACAAGTTGCAGCTTCATATGAATATCCTCCTTAG	Rv – ybeB deletion
2,65	TTACTTTTCTATTTTAGATTATTAAATTTGAGACTTTATTGTGTAGGCTGGAGCTGCTTC	Fw – dcoC deletion
2,66	CGACATCGGTAATGGCAACGGTCATGGATTCCTCCGTGAACATATGAATATCCTCCTTAG	Rv – dcoC deletion
2,67	CAACAAAGGTCGCCAAATTA	Fw – wraB verification
2,68	TTTTCTGCCAGTTTCTCGTC	Rw – wraB verification
2,69	TATCCTGCTGACATTACTCG	Fw – ygaU verification

2,7	AAACATATCCAGCATCACAC	Rw – ygaU verification
2,71	AGGATAGTTATGGTAAACGG	Fw - uspA verification
2,72	TGATGGAATGATGTACTGAG	Rw - uspA verification
2,73	TCCCCTGTAAAGTTATGTCG	Fw - cbpA verification
2,74	TTGCCAGTCGGCGTTATC	Rw - cbpA verification
2,75	GCCCTGTTGGTTTAATATTC	Fw – osmC verification
2,76	TTTAAGGCGAAAAAGTAGGC	Rw - osmC verification
2,77	TCAGAGTAAACCATAACGTG	Fw - ychN verification
2,78	TTATGAAAAAGGCGAGGATG	Rw - ychN verification
2,79	TTAATGATTCCCCTTCCACG	Fw - STM4262 verification
2,8	CCTAACAGAATCGAGCTAAAC	Rw - STM4262 verification
2,81	GTAAAACGGGTTGATTGGTC	Fw - yajD verification
2,82	TCCGGGGCTGAAAAAATG	Rw - yajD verification
2,83	ACATAAGTCCCGGTATCG	Fw - ybeB verification
2,84	TTTGATATCCGCGTTCTTGC	Rv - ybeB verification
2,85	GGATTGGCGATTAGTTTGGT	Fw - dcoC verification
2,86	GGCAGCATATCGTCAAGG	Rw - dcoC verification
